# A Targeted Glycan-Related Gene Screen Reveals Heparan Sulfate Proteoglycan Sulfation Regulates WNT and BMP Trans-Synaptic Signaling

**DOI:** 10.1371/journal.pgen.1003031

**Published:** 2012-11-08

**Authors:** Neil Dani, Minyeop Nahm, Seungbok Lee, Kendal Broadie

**Affiliations:** 1 Department of Biological Sciences and Department of Cell and Developmental Biology, Kennedy Center for Research on Human Development, Vanderbilt University, Nashville, Tennessee, United States of America; 2 Department of Cell and Developmental Biology, Seoul National University, Seoul, Republic of Korea; Harvard Medical School, Howard Hughes Medical Institute, United States of America

## Abstract

A *Drosophila* transgenic RNAi screen targeting the glycan genome, including all N/O/GAG-glycan biosynthesis/modification enzymes and glycan-binding lectins, was conducted to discover novel glycan functions in synaptogenesis. As proof-of-product, we characterized functionally paired heparan sulfate (HS) 6-O-sulfotransferase (*hs6st*) and sulfatase (*sulf1*), which bidirectionally control HS proteoglycan (HSPG) sulfation. RNAi knockdown of *hs6st* and *sulf1* causes opposite effects on functional synapse development, with decreased (*hs6st*) and increased (*sulf1*) neurotransmission strength confirmed in null mutants. HSPG co-receptors for WNT and BMP intercellular signaling, Dally-like Protein and Syndecan, are differentially misregulated in the synaptomatrix of these mutants. Consistently, *hs6st* and *sulf1* nulls differentially elevate both WNT (Wingless; Wg) and BMP (Glass Bottom Boat; Gbb) ligand abundance in the synaptomatrix. Anterograde Wg signaling via Wg receptor dFrizzled2 C-terminus nuclear import and retrograde Gbb signaling via synaptic MAD phosphorylation and nuclear import are differentially activated in *hs6st* and *sulf1* mutants. Consequently, transcriptional control of presynaptic glutamate release machinery and postsynaptic glutamate receptors is bidirectionally altered in *hs6st* and *sulf1* mutants, explaining the bidirectional change in synaptic functional strength. Genetic correction of the altered WNT/BMP signaling restores normal synaptic development in both mutant conditions, proving that altered *trans*-synaptic signaling causes functional differentiation defects.

## Author Summary

Glycans are sugar additions to proteins. Surrounding all eukaryotic cells, secreted and membrane glycans form a glycocalyx that regulates cell–cell signaling. However, the mechanisms controlling glycan-dependent intercellular communication are largely unknown. In the nervous system, glycans play important roles in the development and regulation of synapses mediating intercellular communication. The *Drosophila* neuromuscular junction serves as a genetically tractable synapse in which expression of glycan-related genes can be systematically knocked down to investigate effects on synaptic morphology and function. This study employs a transgenic RNAi screen to characterize the synaptic requirements of 130 glycan-related genes. From this screen, two functionally paired genes (*hs6st* and *sulf1*) that add or remove a sulfate at the 6-O position on heparan sulfate proteoglycans (HSPGs) were identified as being critically important for synaptic functional development. Removal of each gene produces an opposite effect on neurotransmission strength, weakening and strengthening communication, respectively. This mechanism controls the synaptic expression of two HSPGs, which act as co-receptors to control the abundance of anterograde WNT and retrograde BMP signals, which drive intracellular signal transduction pathways regulating gene transcription to control synaptic functional development. This screen serves as a platform for systematic investigation of glycan mechanisms regulating synaptic development.

## Introduction

Glycans coat cell surfaces, and glycosylation decorates secreted molecules of the pericellular space and extracellular matrix (ECM) [1], [2]. It is well known that glycan modifications mediate critical functions of intercellular signaling and regulate interactions of numerous growth factors with the ECM [3], [4]. The synthesis, modification and degradation of glycoconjugates, including O/N-linked glycoproteins, glycosaminoglycan (GAG) proteoglycans and glycan-binding lectins, is controlled by a dedicated cadre of genes [5], [6]. In the nervous system, these glycan-related genes play key roles in development, including neuron fate specification, migration, formation of axon tracts and synapse maturation [7]. At synapses, glycosylated ECM molecules, membrane receptors and outer-leaflet glycolipids together form the highly specialized synaptomatrix interface [4], [8], which interacts with *trans*-synaptic signals to modulate synaptogenesis [9].

A prime example is the classic Agrin proteoglycan, which bears heparan sulfate (HS) chains, O/N-linked glycans and also a glycan-binding lectin domain that binds other glycoconjugates [10], [11], [12]. Reduction of GAG sulfation perturbs the Agrin signaling that drives postsynaptic acetylcholine receptor (AChR) cluster maintenance at the neuromuscular synapse [13]. Likewise, Galbeta1,4GlcNAc and Galbeta1,3GalNAc glycans inhibit Agrin signaling by suppressing muscle specific kinase (MuSK) autophosphorylation, a key step during synaptogenesis [14]. Analogous glycan-dependent mechanisms at the *Drosophila* neuromuscular synapse involve the secreted Mind-the-Gap (Mtg) lectin, which assembles the glycosylated synaptomatrix between presynaptic active zone and postsynaptic glutamate receptor (GluR) domains [15]. This glycan mechanism induces GluR clustering, synaptic localization of integrin ECM receptors, and shapes *trans*-synaptic signaling by controlling ligand/receptor abundance [16], [17], [18]. Thus, many long-term studies in vertebrate and invertebrate genetic models suggest that glycan mechanisms are a core foundation of synapse development.

In the current study, we conducted a broad transgenic RNA interference (RNAi) screen of synaptic glycan function, assaying requirements in both structural and functional development of the *Drosophila* neuromuscular junction (NMJ). We tested 130 genes from 8 functional categories: N-glycan, O-glycan and GAG biosynthesis; glycosyltransferases and glycan modifying/degrading enzymes; glycoprotein and proteoglycan core proteins; sugar transporters and glycan-binding lectins. We found that RNAi-knockdown of genes in all eight categories affects synaptic morphological development, with gene-specific effects on branching, bouton differentiation and synapse area. Likewise, all eight categories regulate synaptic functional development, with gene-specific effects both weakening and strengthening neurotransmission. Interestingly, only a few genes affect both structure and function, suggesting separable roles for glycans in regulating these synaptogenic pathways. The results of this genomic transgenic screen are presented as a platform from which to pursue systematic investigation of glycan mechanisms in synaptic development.

Two genes were selected for screen validation and mechanistic characterization; functionally-paired HS 6-O-endosulfatase (*sulf1*) and HS 6-O-sulfotransferase (*hs6st*). RNAi knockdown and null mutants identically alter synaptic functional development in a bidirectional manner; loss of *sulf1* elevates neurotransmission strength, whereas loss of *hs6st* weakens it. Heparan sulfate proteoglycan (HSPG) targets Dally-like Protein (Dlp) and Syndecan (Sdc) [19], [20] are mislocalized in *sulf1* and *hs6st* null synapses. In other developmental contexts, the sulfation state of these HSPG co-receptors strongly regulates WNT and BMP intercellular signaling [20], [21], [22]. At *Drosophila* synapses, WNT (Wg) is a key anterograde [23], [24] and BMP (Gbb) a key retrograde [25], [26] *trans*-synaptic signal. Consistently, loss of *sulf1* and *hs6st* differentially changes synaptomatrix levels of Wg and Gbb, and downstream signaling into muscle and motor neuron nuclei, respectively. Glutamate release and receptor machinery is thereby bidirectionally altered in the two nulls. Genetic restoration of Wg/Gbb signaling to control levels restores the bidirectional changes in synaptic functional strength and pre-/post- synaptic differentiation in both *sulf1* and *hs6st* nulls. We conclude that extracellular HSPG sulfation state in the synaptomatrix is a point of intersection between WNT/BMP *trans*-synaptic signaling pathways that drive functional development of the neuromuscular synapse.

## Results

### RNAi screen of glycan-related genes identifies multiple synaptogenesis defects

**Figure 1:**
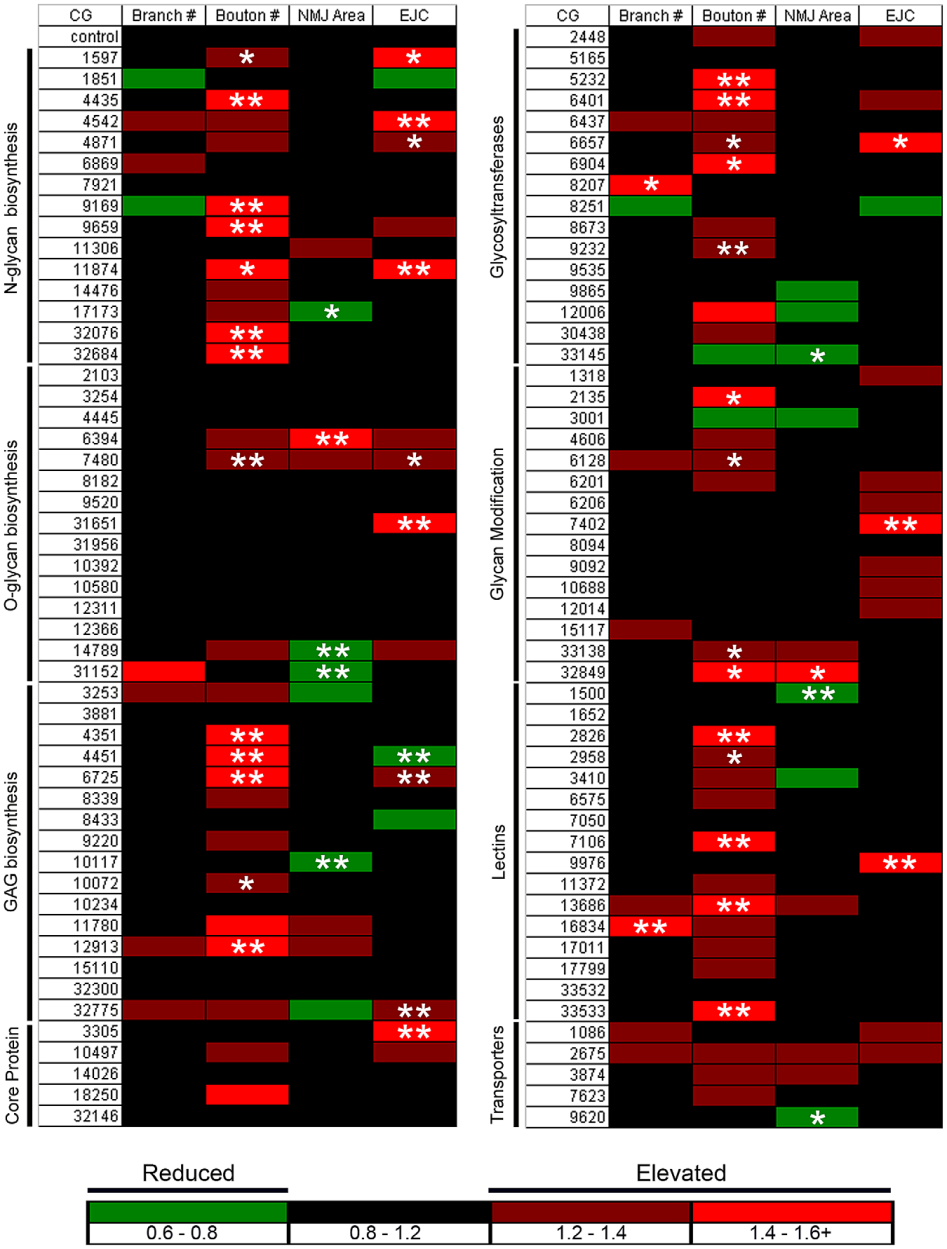
Glycan-related gene RNAi screen for synapse structure/function defects. Transgenic RNAi screen interrogating effects of glycan-related gene knockdown on the morphology and function of the *Drosophila* neuromuscular junction (NMJ) synapse. All VDRC UAS-RNAi lines were crossed to the UH1-GAL4 driver line. Target genes are indicated by *Drosophila* genome CG annotation number and categorized by function. Confocal imaging of co-labeled pre- and postsynaptic markers was used to quantify NMJ architecture, including branch number, bouton number and synaptic area. TEVC electrophysiology was used to quantify evoked excitatory junctional current (EJC) amplitudes. The magnitude of fold changes compared to control (*w^1118^*×UH1-GAL4) is shown on a color scale (see legend below the two columns). Statistical significance was calculated using one-way ANOVA analysis, and displayed as p<0.05 (*), p<0.01 (**).

Synaptic glycans play important roles as ligands, modulators and co-receptors regulating cell-matrix and intercellular communication [3], [27], [28]. Differential glycan distribution on pre- and postsynaptic surfaces, and in the cleft, of numerous protein classes, strongly suggests that glycan mechanisms mediate synaptic structural and functional development [29], [30], [31]. To test the genomic scope of this requirement, we used confocal imaging and electrophysiological recording at the well-characterized *Drosophila* glutamatergic neuromuscular junction (NMJ) [32], [33], [34] to screen the Vienna *Drosophila* RNAi Center (VDRC) library of glycan-related genes [35]. We induced UAS-RNAi knockdown using the ubiquitous UH1-GAL4 driver [15], [36]. We assayed morphological defects by co-labeling for pre- and postsynaptic markers, and assayed functional defects with two-electrode voltage clamp (TEVC) recording of neurotransmission strength. A summary of the screen results is shown in Figure 1. Full numerical results of the screen are shown in Table S1.

Candidate glycan-related genes were identified and classified into eight functional categories using the Kyoto Encyclopedia of Genes and Genomes (KEGG) database [37] (Figure 1). Additional genes were added to the screen based on ortholog identification using the Information Hyperlinked over Proteins (iHOP) database [38]. The candidate gene list was expanded and verified using Flybase [39]. From this list, genes were cross-referenced with available VDRC UAS-RNAi transgenic lines to generate a final candidate list containing 130 genes within eight functionally-defined categories (Figure 1): N-glycan, O-glycan and glycosaminoglycan (GAG) biosynthesis; glycan core proteins (HSPG core proteins/glycoproteins); sugar transporters; glycosyltranferases; glycan modification genes (modification and degradation of glycans); and glycan-binding lectins. On genetic knockdown, 103 lines were viable until the wandering 3^rd^ instar, whereas 27 lines showed developmental lethality at embryonic and early larval stages of development. From the 103 genetic lines characterized by confocal microscopy and TEVC electrophysiology in the 3^rd^ instar (Figure 1), 21 exhibited pupal stage developmental lethality. Interestingly, >50% of pupal lethal lines displayed statistically significant defects in NMJ synaptic morphology and function.

For all 103 larval-viable lines, synapse morphology and function was quantified at the wandering 3^rd^ instar NMJ (Figure 1; Table S1). Each UAS-RNAi line driven by UH1-GAL4 in the *w^1118^* background was compared to the genetic control of *w^1118^* crossed to UH1-GAL4 (UH1-GAL4×*w^1118^*) [35]. All morphological and functional assays were done blind to genotype, with values reported as fold-change compared to genetic control, as well as statistical significance calculated using one-way ANOVA analyses (see color scheme; P<0.05 (*), P<0.01 (**); Figure 1). The data represents ≥6 NMJs from ≥3 animals from every genotype. Synapse morphology was imaged by co-labeling with presynaptic marker anti-horse radish peroxidase (HRP) and postsynaptic marker anti-Discs Large (DLG). A synaptic bouton was defined as a varicosity of ≥2 µm in minimum diameter labeled by both HRP and DLG, and a synaptic branch was defined as a process containing at least two boutons [40]. NMJ branch number was the least affected morphological parameter, with only 2 of 103 genes showing a statistically significant change (Figure 1). Many more genes were involved in bouton development. All 27 genes showing a statistically significant change compared to genetic control exhibited elevated bouton numbers (Figure 1), suggesting that glycan mechanisms primarily limit morphological growth. Synapse area was determined by outlining the terminal area labeled by DLG using the thresholding function in ImageJ. The majority of gene knockdown conditions showed a decrease in NMJ area compared to control (Figure 1). 7 RNAi lines exhibited a statistically significant decrease in area, whereas only 2 lines exhibited a statistically significant increase in synaptic area. All raw values of measured morphological parameters are included in Table S1.

To assay functional differentiation, the motor nerve was stimulated with a suction electrode while the evoked excitatory junctional current (EJC) was recorded in the muscle (Figure 1) [41]. Nerve stimulation was applied at 4 V for 0.5 ms at a frequency of 0.2 Hz, with the muscle clamped at −60 mV. EJC amplitudes were calculated from recorded traces in the ubiquitously-driven RNAi lines (*w^1118^* background) compared to the *w^1118^*; UH1-GAL4*/+* control. Recordings were obtained from ≥3 independent trials for each RNAi knockdown condition. All electrophysiological screening was done blind to genotype, with values reported as fold-change and statistical significance calculated by one-way ANOVA analyses (see color scheme; P<0.05 (*), P<0.01 (**); Figure 1). Genes from all eight glycan classes were identified to produce changes in neurotransmission strength upon genetic knockdown. For the 103 larval-viable lines tested, 26 lines showed a trend towards increased transmission strength, and 12 were statistically elevated compared to genetic control (Figure 1). 4 gene knockdowns showed a trend towards decreased transmission strength, of which only 1 line reached statistical significance. 73 of the 103 lines tested showed no change in functional strength (Figure 1). Interestingly, only 6 RNAi lines showed statistically significant effects on both NMJ morphology parameters and EJC amplitude: CG1597, CG6657, CG7480, CG4451, CG6725 and CG11874 (Figure 1). This suggests that glycan effects on synapse morphological and functional development are largely separable. All raw values of EJC measurements are included in Table S1.

To validate results, a secondary screen was conducted using independent RNAi lines obtained from the VDRC and Harvard TRiP collections (Table S2). Of the 44 genes that showed morphological and functional defects in the primary screen, 33 were retested using independent RNAi lines, with the others lacking available secondary lines from any source. Using the same screen of morphological and functional characterization, we determined that ∼80% of retested secondary lines showed the reported structural (bouton number) and functional (EJC) phenotypes consistent with primary screen (Table S2). These primary and secondary RNAi screen results now represent a resource for the systematic characterization of glycan mechanisms underlying synaptic structural and functional development. Screen results were further studied by comparing synaptogenesis phenotypes of RNAi knockdown with defined genetic nulls for two genes, CG6725 and CG4451, from the glycosaminoglycan biosynthesis class (Figure 1). The RNAi screen of functional strength as measured by EJC amplitudes indicated opposite effects for these two lines, with CG6725 (RNAi-*sulf1*) knockdown exhibiting an increase in transmission strength and CG4451 (RNAi-*hs6st*) knockdown producing a decrease (Figure 1). Along with our goal to identify interesting glycan-related genes involved in synapse development, we show here characterization of null alleles of two genes obtained from screen results and define the associated mechanisms driving the bidirectional regulation of synaptic functional development.

### Synaptogenesis is bidirectionally regulated by paired *sulf1* and *hs6st* genes

The RNAi screen identified two functionally-paired genes, *sulf1* (CG6725) and *hs6st* (CG4451), with similar effects on morphological development but opposite effects on synaptic functional differentiation (Figure 1). Our goal was to use these genes as a test case from the completed glycan screen, by assaying phenotypes in recently characterized null mutants of both genes [42], [43]. The gene products Sulfated (Sulf1), an HS 6-endosulfatase, and Hs6st, an HS 6-O-sulfotransferase, drive opposing changes in sulfation state of the same C_6_ carbon of the repeated glucosamine unit in GAG modified heparan sulfate proteoglycans [43], [44]. Viable null mutants are available for both genes, e.g. *sulf1* (*sulf1^Δ1^*) and *hs6st* (*hs6st^d770^*) [42], [43], but requirements have never been assayed in the nervous system or neuromusculature. We therefore first compared phenotypes of RNAi knockdown and null alleles at the NMJ synapse by confocal imaging of synaptic morphogenesis and TEVC recording of synaptic functional neurotransmission.

Using double-labeling for HRP (presynaptic) and DLG (postsynaptic), NMJ structural parameters including bouton number, branch number and synaptic area were quantified in *sulf1* and *hs6st* null alleles. The mutant results closely recapitulated the RNAi knockdown findings from the screen (Table S1). To consistently compare RNAi and null mutant conditions, both animal groups were simultaneously reared and processed to visualize the NMJ (Figure S1). Structural quantification showed an increased bouton number with RNAi-mediated *sulf1* knockdown (*sulf1-*RNAi×UH1-GAL4; 36.4±1.6, n = 10) and *hs6st* knockdown (*hs6st-*RNAi×UH1-GAL4; 35.1±1.96, n = 10) compared to the transgenic control (*w^1118^*×UH1-GAL4; 21.9±1.84, p<0.001, n = 10; Figure S1A, S1B). Consistently, increased bouton number was observed in both *sulf1* (31.9±1.37, n = 10) and *hs6st* (36.25±2.58, n = 8) null mutants compared to genetic control (*w^1118^, 19.3*±1.69, p<0.001, n = 10; Figure S1C, S1D). In contrast, no significant change in branch number was exhibited with *sulf1* knockdown (3.22±0.28, p>0.05, n = 9) or *hs6st* knockdown (3.22±0.22, p>0.05, n = 9) compared to control (*w^1118^*×UH1-GAL4; 2.64±0.06, n = 11). Similarly, no significant change was observed in the synaptic branch number in *sulf1* (2.8±0.33, p = 0.27, n = 10,) and *hs6st* (3.63±0.38, p = 0.115, n = 10) nulls compared to control (*w^1118^*; 3.4±0.46, n = 8). Further, there was no significant difference in synaptic area in *sulf1* (138.16±5.82, p>0.05, n = 10,) and *hs6st* (138.48±13.38, p>0.05, n = 8,) mutants compared to the control (*w^1118^*; 118.04±8,38, n = 10), however a slight increase in synaptic area was observed in *sulf1* knockdown (178.68±10.64, p<0.05, n = 9), while no change was observed for *hs6st* knockdown (164±8.47, p>0.05, n = 10) as compared to control (*w^1118^*×UH1-GAL4; 134.57±11.95, n = 10). Based on these imaging studies, we conclude morphological differences in synaptic architecture observed in both *sulf1* and *hs6st* null allele conditions are consistent with both RNAi knockdown conditions.

**Figure 2:**
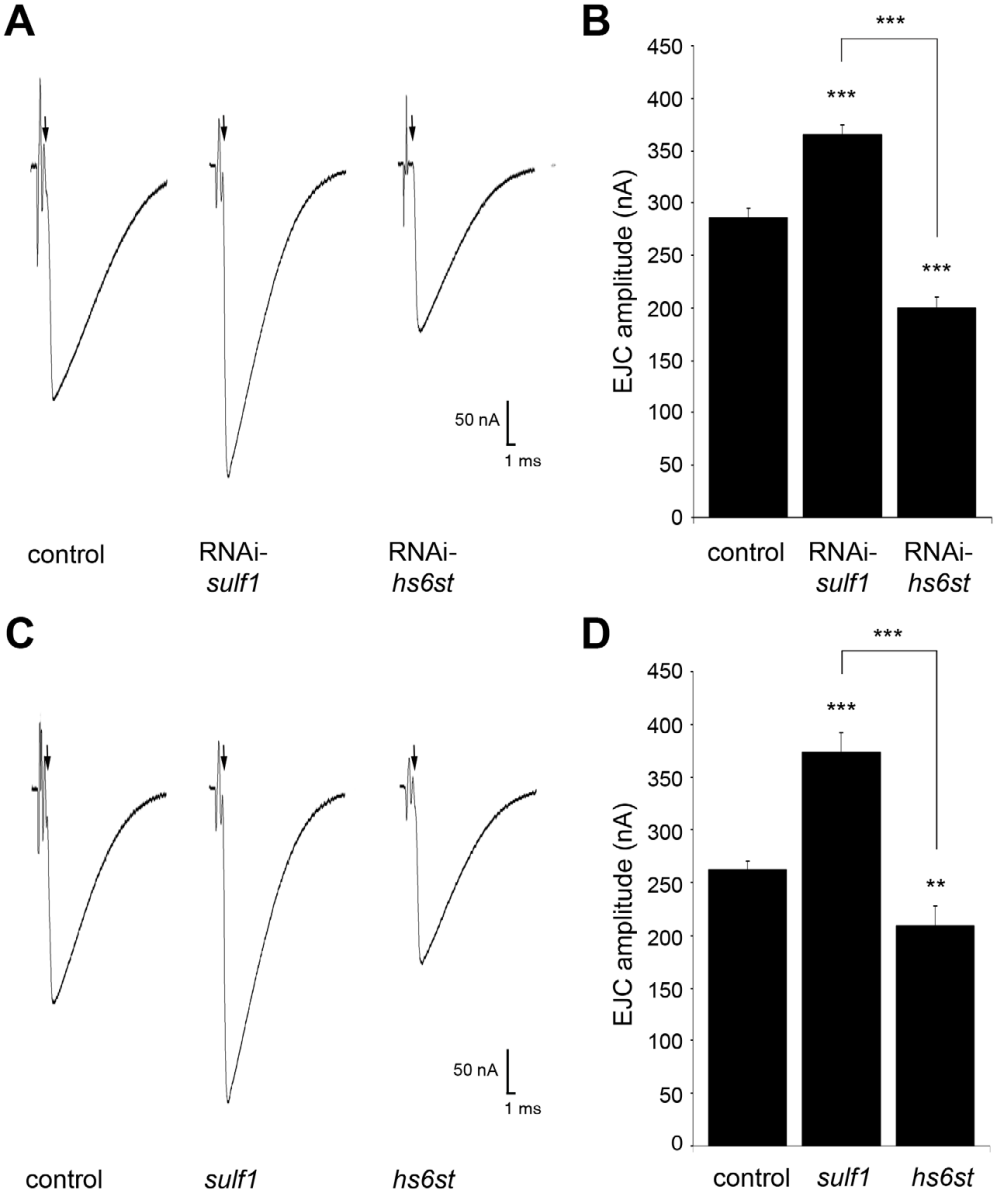
Loss of *sulf1*/*hs6st* causes opposite effects on transmission strength. (A) Representative excitatory junctional current (EJC) traces from control (*w^1118^*×UH1-GAL4), *sulf1* RNAi (UH1-GAL4×UAS-CG6725) and *hs6st* RNAi (UH1-GAL4×UAS-CG4451). The nerve was stimulated (arrows) in 1.0 mM external Ca^2+^, with TEVC records (−60 mV holding potential) from muscle 6 in segment A3. Each trace averaged from 10 consecutive recordings. (B) Quantified mean EJC amplitudes (nA) for the three genotypes shown in panel A. (C) Representative traces from control (*w^1118^*), *sulf1^Δ1^* and *hs6st^d770^* null alleles under the same conditions described in panel A. (D) Quantified mean EJC amplitudes (nA) for the three genotypes shown in panel C. Sample sizes are at least 11 animals per indicated genotype. Statistically significant differences calculated using student's t-test, ** p<0.01, *** p<0.001. Error bars indicate S.E.M.

Functional development was next tested with electrophysiological recording to compare RNAi and null mutant phenotypes (Figure 2). Representative TEVC records are shown as an average of 10 consecutive nerve stimulus responses in 1.0 mM extracellular Ca^2+^ for each transgenic genotype in Figure 2A; *sulf1* knockdown (UH1-GAL4×*sulf1-*RNAi), *hs6st* knockdown (UH1-GAL4×*hs6st-*RNAi) and genetic control (UH1-GAL4×*w^1118^*). There was a striking ∼80% difference in EJC amplitude between *sulf1* and *hs6st* knockdown conditions, with *sulf1* elevated by ∼30% and *hs6st* reduced by ∼30% compared to control. Quantification of EJC amplitudes showed both knockdown conditions to be highly significantly different from control and each other (control, 286.22±8.56 nA; *sulf1*-RNAi, 365.01±9.502 nA, p<0.001; *hs6st-*RNAi, 199.19±11.84 nA, p<0.001; *sulf1*-RNAi vs. *hs6st-*RNAi, p<0.001; Figure 2B). These opposite effects on neurotransmission strength were confirmed in characterized null alleles for both genes [42], [43]. Representative traces from *sulf1^Δ1^* and *hs6st^d770^* null mutants compared to *w^1118^* control are shown in Figure 2C. Quantification of EJC amplitudes showed null mutants to be highly significantly different from control and each other (*w^1118^*, 256.14±7.38 nA; *sulf1^Δ1^,* 372.86±18.49 nA, n = 11, p<0.001; *hs6st*, 209.66±13.44 nA, n = 14, p<0.01; *sulf1^Δ1^* vs. *hs6st*, p<0.001; Figure 2D). These results were confirmed in an independent *sulf1* null allele (*sulf1^ΔP1^*), which shows comparable elevation compared to control (*w^1118^*, 244.91±9.04 nA; *sulf1^ΔP1^*, 282.28±13.59, p<0.05, n = 22), as well as the *hs6st* null (*hs6st^d770^*) over deficiency (Df(3R)ED6027), which shows comparable depression compared to control (*w^1118^*, 256.14±7.38 nA; *hs6st*/Df(3R)ED6027, 224.06±7.65 nA, p<0.05, n = 18). These results reveal a critical role for *sulf1* and *hs6st* genes in synaptic functional development.

Given the functionally-paired nature of *sulf1* and *hs6st* activities on 6-O-S modification, and the epistatic function of *hs6st* to *sulf1*, we predicted that knocking both genes down would produce a phenotype similar to knockdown of *hs6st* alone. Consistently, *hs6st* and *sulf1* double knockdown produced EJC amplitudes significantly lower than control (*w^1118^*×*hs6st*-RNAi; *sulf1*-RNAi (control), 225.17±6.28 nA, n = 12; *hs6st*-RNAi, *sulf1*-RNAi×UH1-GAL4, 198.22±9.77 nA, n = 15, p<0.05; Figure S2). Cell-specific knockdown in neural (*elav*-GAL4), muscle (24B-GAL4) and glia (*repo*-GAL4) also support the observed opposite effects in neurotransmission strength. With *sulf1* knockdown in muscle, EJC amplitude was significantly elevated compared to control (*w^1118^*×*sulf1*-RNAi (control), 199.97±21.86 nA; 24B-GAL4×*sulf1*-RNAi (knockdown), 222.88±25.78 nA, p<0.01, n = 10), but no change occurred with neural knockdown (*elav*-GAL4×*sulf1*-RNAi, 196.09±25.08 nA, p = 0.72, n = 10) or glial knockdown (*repo*-GAL4×*sulf1*-RNAi, 208.40±32.45 nA, p = 0.53, n = 7). Moreover, only neural knockdown of *hs6st* caused a decrease in EJC amplitude (*w^1118^*×*hs6st*-RNAi (control), 211.496±22.142 nA, *elav*-GAL4×*hs6st*-RNAi (knockdown), 184.68±28.97 nA, p<0.05, n = 16), while no change occurred with muscle knockdown (24B-GAL4×*hs6st*-RNAi, 209.92±24.74 nA, p = 0.88, n = 9) or glial knockdown (*repo*-GAL4×*hs6st*-RNAi, 216.38±37.80 nA, p = 0.32, n = 7). We conclude that HSPG sulfation state strongly modulates NMJ functional development, with contributions from both motor neuron and muscle, but not glia. The clear next step was to test for differences in the localization and abundance of synaptic HSPG targets known to regulate NMJ synaptogenesis.

### HSPG abundance at the synaptic interface is dependent on *sulf1* and *hs6st*

**Figure 3:**
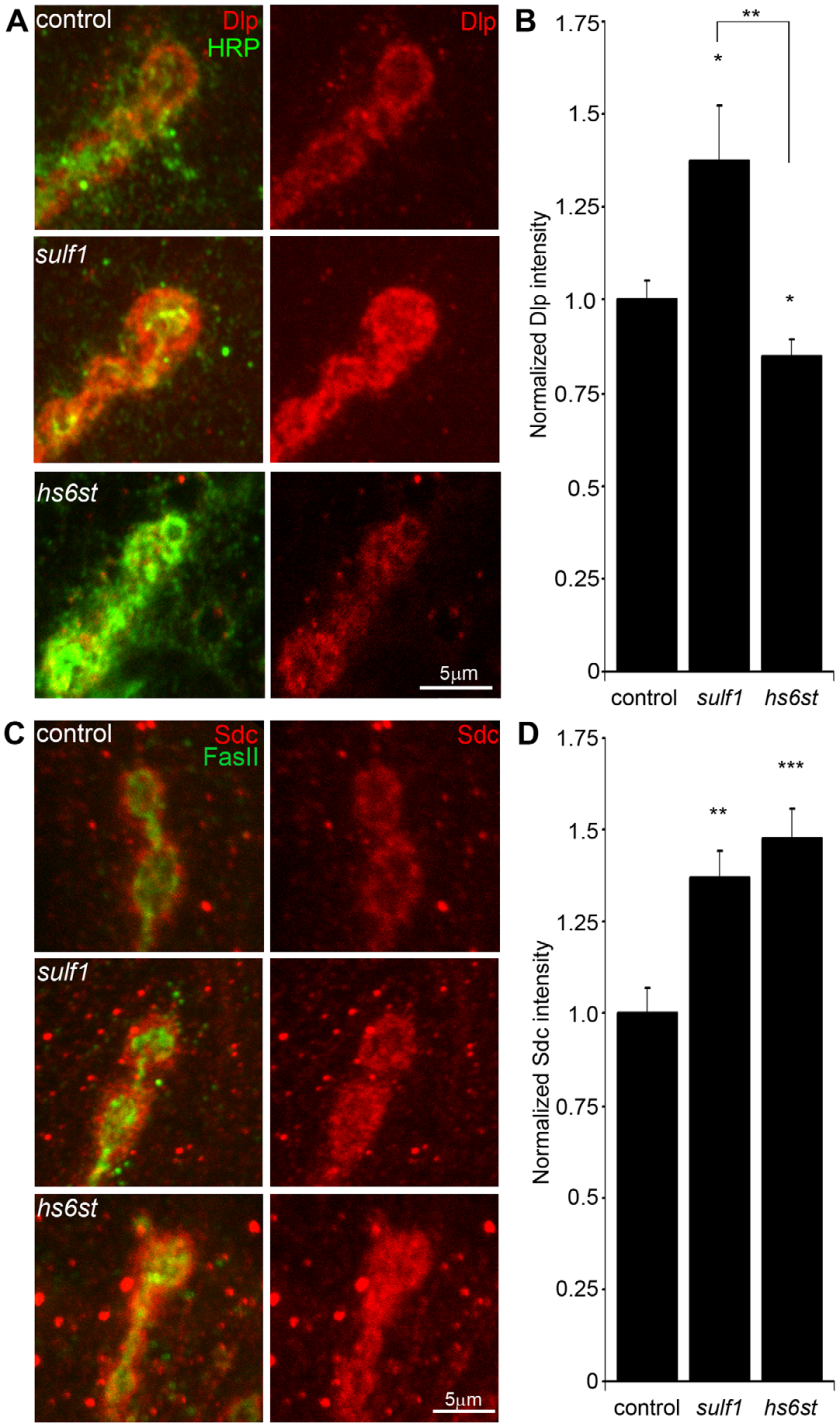
Synaptic HSPG co-receptor abundance is modified by 6-O-S sulfation. (A) Representative NMJ synaptic boutons imaged from control (*w^1118^*), *sulf1* and *hs6st* nulls, probed with presynaptic neural marker anti-HRP (green) and Dally-like (Dlp; red). Right: Dlp distribution without the HRP signal is shown for clarity. (B) Quantification of mean fluorescent intensity levels of anti-Dlp labeling normalized to the HRP co-label at the muscle 6 NMJ, normalized to genetic control. (C) Boutons labeled with neural marker anti-Fasciclin II (FasII, green) and anti-Syndecan (Sdc, red). Right: Sdc distribution is shown alone for clarity. (D) Quantification of the mean fluorescent intensity levels of anti-Sdc labeling at the muscle 6 NMJ, normalized to genetic control. Sample sizes are at least 12 independent NMJs of at least 7 animals per indicated genotypes. Statistically significant differences calculated using student's t-test, * p<0.01, ** p<0.01, ***p<0.001. Error bars indicate S.E.M.

Both GPI-anchored HSPG glypican Dally-like (Dlp) and transmembrane HSPG Syndecan (Sdc) are clearly expressed at the *Drosophila* NMJ (Figure S3), where they are known to regulate synaptogenesis [45]. We detect no enrichment of the secreted HSPG perelcan (Trol) at the NMJ, although it is abundantly expressed in the motor nerve leading up to the synaptic terminal and present in lower levels throughout the muscle (Figure S4). We therefore hypothesized that membrane-associated Dlp and Sdc HSPGs are targeted by *sulf1* and *hs6st* activity to regulate their synaptic distribution and/or function. To test this hypothesis, we assayed both Dlp and Sdc under non-permeabilized, detergent-free conditions to examine their cell surface expression at the NMJ synaptic interface of *sulf1* and *hs6st* null mutants compared to control. These data are summarized in Figure 3.

In the genetic background control (*w^1118^*), Dlp shows a punctate expression pattern strongly concentrated in a halo-like array around the anti-HRP labeled presynaptic membrane (Figure 3A, top; Figure S3). In *sulf1* mutants there was a clear and consistent increase in Dlp abundance, with more numerous and intense punctae at the synaptic interface surrounding NMJ boutons, while at *hs6st* mutant synapses there was an opposing decrease in Dlp abundance (Figure 3A). This bidirectional and differential effect on Dlp abundance was quantified as fluorescence intensity normalized to the internal HRP labeling control. There was a significant Dlp increase in *sulf1* compared to control (∼40% elevated over control; p<0.05; n = 11), and a significant Dlp decrease in the *hs6st* null synapse (∼15% reduced compared to control; p<0.05; n = 11; Figure 3B). Importantly, the difference between *sulf1* and *hs6st* nulls was very highly significant (p<0.001). In comparison, cell surface Sdc labeling also showed a dense halo-like localization around NMJ synaptic boutons labeled with cell adhesion marker Fasciclin II (FasII; Figure 3C; Figure S3). Synaptic Sdc labeling intensity was consistently greater in both *sulf1* and *hs6st* nulls compared to control (Figure 3C). Quantification of fluorescence intensity normalized to HRP revealed that Sdc abundance was greatly increased in *sulf1* null synapses compared to control (∼35% elevated over control; p<0.01; n = 17) and, to a greater degree, also in *hs6st* nulls (∼50% elevated over control; p<0.001; n = 12; Figure 3D). Thus, both Dlp and Sdc HSPGs are strongly altered in *sulf1* and *hs6st* null NMJ synapses, with Dlp bidirectionally misregulated and Sdc differentially elevated in the two mutant conditions.

HSPGs act as co-receptors for WNT and BMP intercellular signaling ligands in many developmental contexts, acting to modulate extracellular ligand abundance and downstream signaling [46], [47]. *Drosophila* WNT Wingless (Wg) distribution and signaling is known to be modulated by Dlp, which retains Wg at the cell surface in a mechanism that is enhanced by HS GAG chains [48]. Specifically, Wg ligand abundance and signaling activity along the dorso-ventral axis of the developing *Drosophila* wing disc is elevated in *sulf1* mutants [22]. Likewise, BMP ligands in other cellular contexts are closely regulated by HSPG co-receptors [20]. Specifically, Dlp has been suggested to similarly regulate *Drosophila* BMP Glass Bottom Boat (Gbb) [20]. We therefore hypothesized that altered HSPG co-receptors Dlp and/or Sdc in *sulf1* and *hs6st* null synapses regulate Wg and Gbb abundance to drive differentially altered *trans*-synaptic signaling across the synaptic cleft.

### HSPG sulfation regulates abundance of WNT/BMP *trans*-synaptic ligands

**Figure 4:**
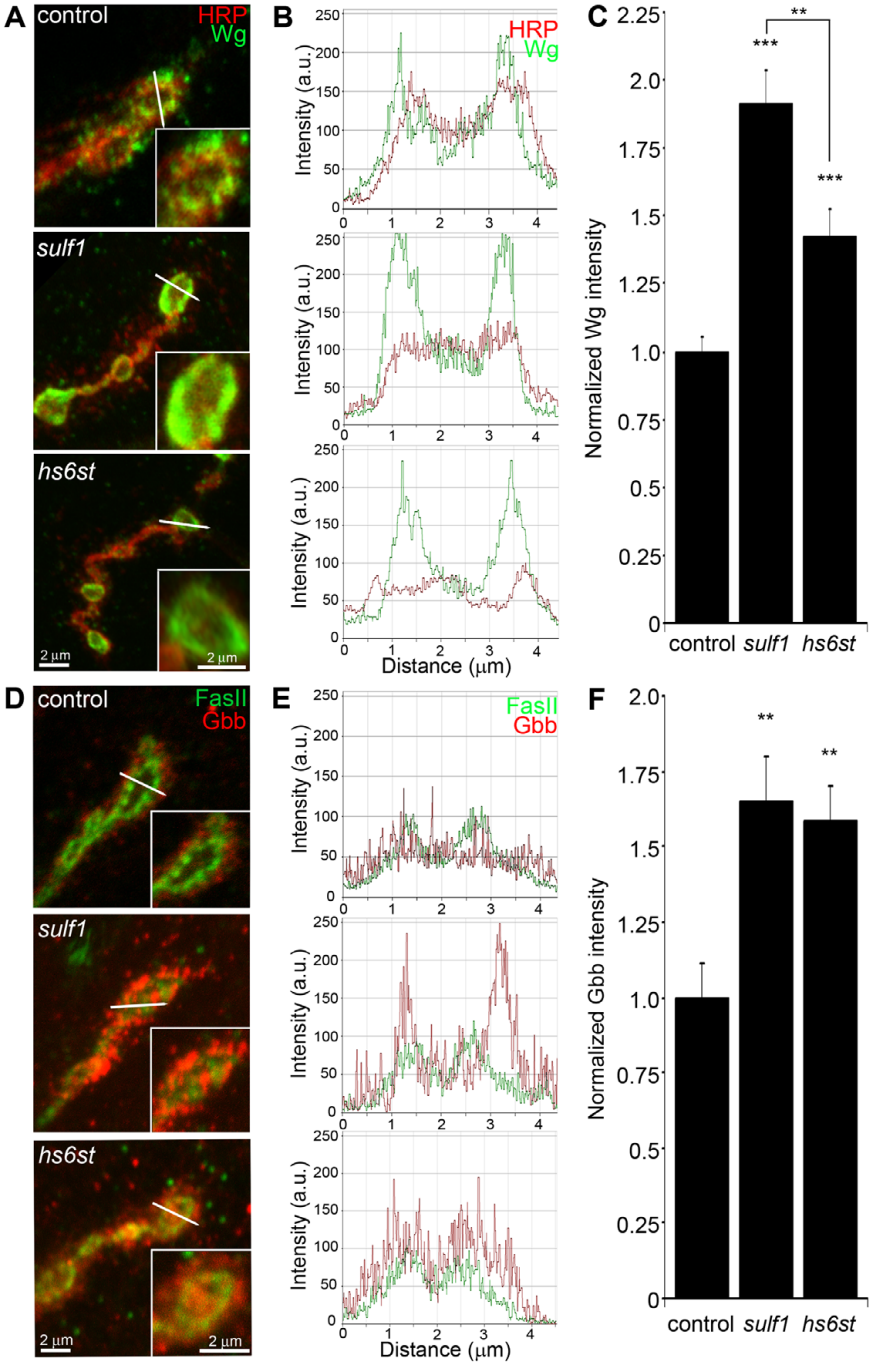
Synaptic WNT and BMP ligand abundance is modified by 6-O-S sulfation. Images show muscle 6 NMJ in segment A3 probed in non-detergent conditions, so that only extracellular protein distributions are detected. The white lines indicate cross-section planes for spatial measurements. Insets indicate single synaptic boutons at higher magnification. (A) Representative NMJ boutons from control (*w^1118^*), *sulf1* and *hs6st* null genotypes, labeled for presynaptic anti-horseradish peroxidase (HRP, red) and anti-wingless (Wg, green). (B) Extracellular distribution of Wg across the diameter of a synaptic bouton. The Y-axis indicates intensity and the X-axis shows distance in microns. The HRP intensity profile is indicated in red; Wg intensity is shown in green. (C) Quantification of Wg mean intensity levels normalized to the HRP co-label, and to genetic control. Sample sizes are at least 15 animals per indicated genotypes. (D) Representative synaptic boutons labeled with presynaptic anti-Fasciclin II (FasII; green) and anti-Glass Bottom Boat (Gbb; red). (E) Gbb distribution across the diameter of a synaptic bouton. Y-axis indicates intensity and the X-axis shows distance in microns. FasII intensity profile is indicated in green; Gbb intensity is shown in red. (F) Quantification of Gbb mean intensity levels normalized to genetic control. Sample sizes are at least 11 independent NMJs of at least 7 animals per indicated genotypes. Statistically significant differences calculated using student's t-test and Mann-Whitney test for non-parametric data, ** p<0.01, *** p<0.001. Error bars indicate S.E.M.

Classical WNT and BMP morphogens act locally at synapses to fine tune synaptogenesis [49], [50]. At the *Drosophila* NMJ, the WNT Wg is well-characterized as an anterograde *trans*-synaptic signal modulating synaptogenesis [23], [24], [51]. Similarly, the BMP Gbb is well-characterized as a retrograde signal driving synaptic development [25], [26], [52]. A third *trans*-synaptic signaling pathway, presynaptically-secreted Jelly Belly (Jeb) to postsynaptic Alk receptor [17], has no known interaction with HSPGs and therefore would not be expected to be affected in *sulf1* and *hs6st* nulls, providing a comparison for specificity. To test the hypothesis that the observed alterations of HSPG co-receptor abundance will drive specific changes in WNT and BMP intercellular pathways, we labeled NMJ synapses with antibodies under non-permeablized conditions to reveal extracellular *trans*-synaptic signaling ligands (Figure S5), and compared protein abundance and distribution in controls, *sulf1* and *hs6st* null mutants. The data are summarized in Figure 4.

NMJ synapses were first labeled with Wg antibody (green) together with anti-HRP (red) to label the presynaptic membrane (Figure 4A). In control animals (*w^1118^*), external Wg localized at large type Ib synaptic boutons in a dynamic pattern of punctuate distribution at the synaptic interface between motor neuron and muscle (Figure 4A, top; Figure S5). In *sulf1* and *hs6st* mutants, Wg was consistently elevated and concentrated uniformly in the extracellular domain adjacent to, and overlapping with, the anti-HRP-labeled presynaptic membrane (Figure 4A, middle and bottom). The elevated Wg levels in mutants were clearly observed at the level of individual synaptic boutons, as shown in the magnified insets in Figure 4A. To examine changes in Wg spatial distribution, cross-sectional planes were examined in single confocal line scans through the diameter of individual synaptic boutons (Figure 4A, white lines). Representative distribution plots for membrane-marker HRP (red) and external Wg (green) are shown in Figure 4B. In all genotypes, extracellular Wg was closely associated with the HRP-labeled presynaptic membrane, but both *sulf1* and *hs6st* nulls displayed a consistent increase in Wg label intensity and broadening of the spatial domain occupied by the secreted Wg ligand (Figure 4B, middle and bottom). To quantify changes in extracellular Wg abundance, the mean fluorescent signal intensity was normalized to the internal HRP co-label, and then normalized to analogous control intensity ratios. In *sulf1^Δ1^* nulls, there was very highly significant elevation of Wg compared to control (∼90% increased; p<0.001; n = 16; Figure 4C). A similar increase was observed in the independent *sulf1^ΔP1^* null (p<0.001; n = 11). The *hs6st* null displayed a smaller significant increase in Wg abundance (∼40% increased; p<0.001; n = 15; Figure 4C), which was again recapitulated in *hs6st* null over deficiency (Df(3R)ED6027) condition. Importantly, Wg abundance is differentially elevated in *sulf1* vs. *hs6st* mutants (p<0.01, Figure 4C).

To test whether the *sulf1/hs6st* mechanism might coordinately regulate multiple *trans*-synaptic signals, we next assayed the BMP Gbb, a muscle-derived retrograde signal [25]. A barrier to previous Gbb analyses has been the absence of an anti-Gbb antibody. We therefore generated a specific anti-Gbb antibody for this study (see Methods). As above, labeling was done under non-permeabilized conditions to reveal only the extracellular Gbb, together with labeling for HRP or the cell adhesion molecule marker FasII to reveal the presynaptic membrane (Figure S5). In the control (*w^1118^*), extracellular Gbb concentrated in a ring of punctate domains around boutons (Figure 4D, top). Gbb was similarly punctate in *sulf1* and *hs6st* nulls, but consistently more extensive and denser (Figure 4D, middle and bottom; see magnified insets). To examine Gbb spatial distribution, cross-sectional planes of confocal line scans were made through individual synaptic boutons (Figure 4D, white lines). Representative plots for FasII (green) and Gbb (red) show extracellular Gbb closely associated with the FasII-labeled presynaptic membrane in all genotypes (Figure 4E). However, *sulf1* and *hs6st* nulls consistently displayed increased Gbb intensity and broadened expression compared to the control. Upon quantifying signal intensity of Gbb normalized to HRP co-label, *sulf1^Δ1^* exhibited a significantly higher Gbb abundance than control (65% increased; p<0.01; n = 12; Figure 4F). The independent *sulf1^ΔP1^* null allele showed a similar increase (p<0.001; n = 12). The *hs6st* null also showed Gbb elevation compared to control (59% increased; p<0.01; n = 11; Figure 4E), which was confirmed in *hs6st* null over deficiency (Df(3R)ED6027; p<0.05; n = 23).

To test further whether extracellular Wg and Gbb abundance was sensitive to the sulfation state of GAGs, a biochemical approach was next used to determine effects on Wg and Gbb *trans-*synaptic signals (Figure S6). Specifically, NMJs were acutely exposed to heparin, the most sulfated form of GAG [53], and then synaptic Wg and Gbb abundance was measured by immunolabeling as above. We found that both *trans-*synaptic signals were rapidly altered by heparin incubation in a dose-dependent manner. Specifically, incubation with increasing concentrations of heparin caused a reciprocal decrease in Wg labeling intensity in the NMJ synaptic domain (Figure S6A, S6C), with a significant decrease first detected with 0.315 mg/ml heparin incubation (∼50% less than control, p<0.01, n = 4). Interestingly, increasing heparin concentrations caused a parallel increase in Gbb abundance in the NMJ synaptic domain (Figure S6B, S6C) in a dose-dependent manner, with significant increases again first detected at 0.315 mg/ml heparin (∼25% greater than control, p<0.05) and rising further at 0.625 mg/ml heparin (∼40% greater than control, p<0.001). These results indicate that HSPG sulfation state does indeed affect *trans-*synaptic signal abundance, supporting the observed alterations in Wg and Gbb abundance in mutants of heparan sulfate modifying genes, *sulf1* and *hs6st.*

To examine effects on other *trans-*synaptic signaling pathways in the *sulf1* and *hs6st* mutant synapses, we also assayed for changes in Jeb [17] and FGF [17] signaling. In both control and mutants, extracellular Jeb labeling was tightly associated with NMJ type Ib boutons and, like other *trans-*synaptic ligands, occupied an extracellular domain closely associated with the presynaptic membrane (Figure S7A). However, in stark contrast to Wg and Gbb ligands in the same extracellular synaptomatrix domain, no change was observed in Jeb abundance or spatial distribution in *sulf1* null (p = 0.99, n = 10) or *hs6st* null (p = 0.36, n = 8) compared to control (*w^1118^*) NMJ synapses (Figure S7B). FGF signaling is also well established to be affected by HSPGs [54], and one pioneering study has investigated roles for FGF signaling at the *Drosophila* NMJ [55]. The probe used in the previous study was an antibody against the FGF receptor Heartless (Htl) [56]. Using this antibody, we confirmed that the Htl receptor beautifully localizes to NMJ boutons to mediate FGF signaling (Figure S8A). However, Htl receptor synaptic abundance and distribution was very similar for the *sulf1* (p = 0.89, n = 9) and *hs6st* (p = 0.69, n = 7) mutants compared to control (*w^1118^*) (Figure S8B). Unfortunately, no antibody probes are available for *Drosophila* FGF ligands, so these signals have not yet been queried. Together, these results show that both WNT (Wg) and BMP (Gbb) ligand abundance is coordinately upregulated by the *sulf1* and *hs6st* mechanism at the NMJ synapse, but that a spatially overlapping signaling ligand (Jeb) and at least FGF receptor expression are unaffected. These results strongly predict that Wg and Gbb *trans*-synaptic signaling controlled by *sulf1* and *hs6st* activity regulates synaptic functional development.

### *Trans*-synaptic WNT/BMP signaling is regulated by HSPG sulfation

Wg and Gbb serve as anterograde and retrograde *trans*-synaptic signals, respectively, activating cognate receptors to initiate downstream signaling cascades and nuclear import pathways in muscles and motor neurons, respectively [24], [26], [50], [51]. The anterograde Wg signal drives dFrizzled-2 (dFz2) receptor internalization in the postsynaptic domain followed by cleavage of the receptor C-terminus, which then enters the muscle nuclei [57]. The muscle-derived retrograde Gbb signal activates presynaptic receptors to drive phosphorylation of the Mothers Against Decapentaplegic (Mad) transcription factor, and then P-Mad enters the motor neuron nuclei to regulate transcription [25], [26], [58]. Given the differential change in both HSPG co-receptor and Wg/Gbb ligand abundance in *sulf1* vs. *hs6st* mutants, we hypothesized that these signaling pathways would be differentially affected during synaptogenesis. We therefore quantitatively assayed the paired muscle and motor neuron nuclear import pathways to determine whether and how *trans*-synaptic signaling may be modulated by *sulf1* and *hs6st* at the NMJ synapse.

**Figure 5:**
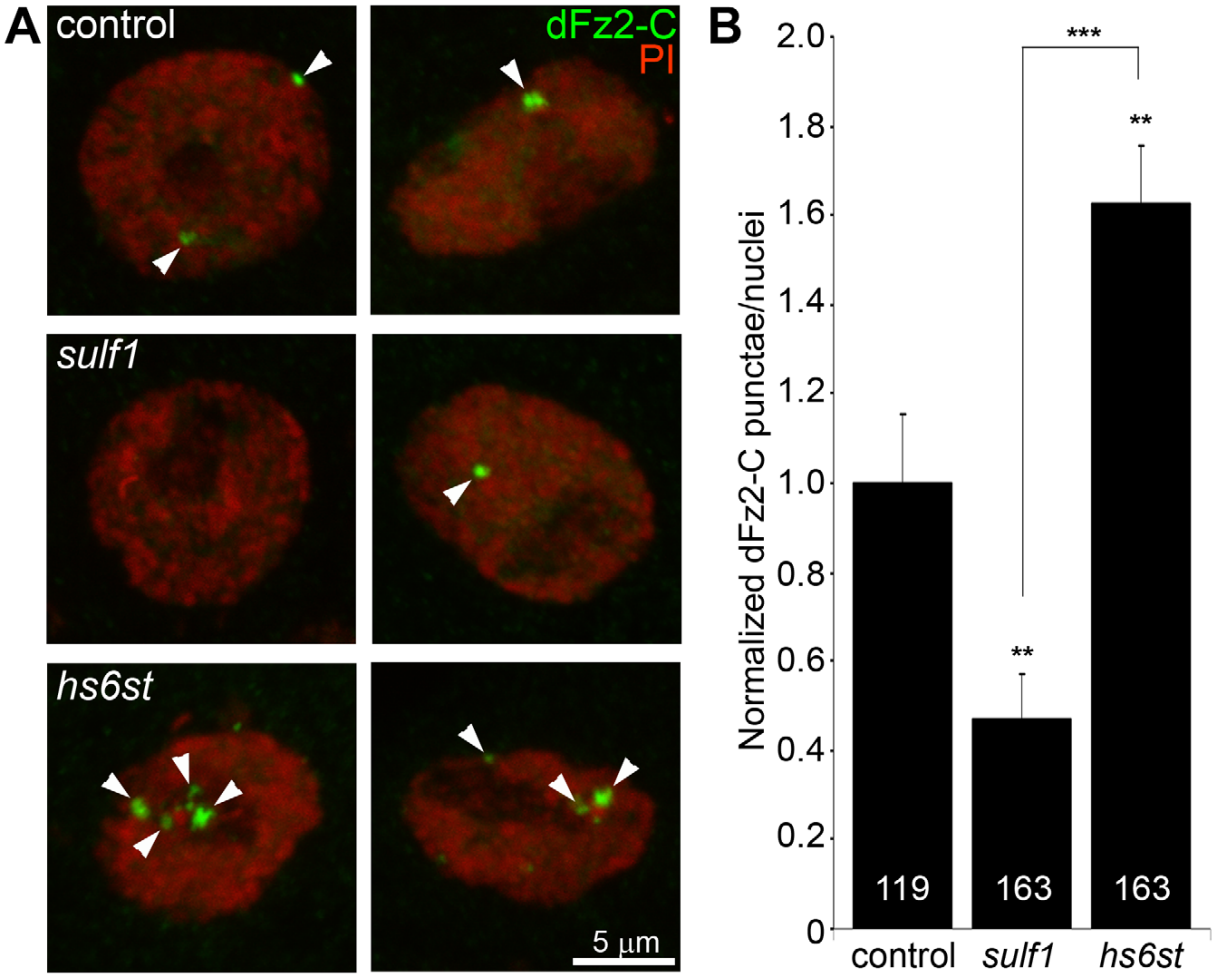
Loss of *sulf1* and *hs6st* causes opposite effects on WNT signaling. (A) Representative images of muscle nuclei from control (*w^1118^*), *sulf1* and *hs6st* nulls, labeled with nuclear marker propidium iodide (PI, red) and for the C-terminus of the Wingless receptor Frizzled 2 (dFz2-C, green). Arrows indicate punctate dFz2-C nuclear labeling. Nuclei shown from muscle 6 in segment A3. (B) Quantification of the number of dFz2-C punctae per nuclei, normalized to genetic control. The total number of nuclei analyzed is indicated in each column; 119 for control (*w^1118^*) and 163 nuclei each for *sulf1* and *hs6st* null mutants. Sample sizes are ≥9 animals per indicated genotypes. Statistically significant differences calculated using student's t-test; ** p<0.01 *** p<0.001. Error bars indicate S.E.M.

Characterized antibodies specifically recognizing the N- and C-termini of the Wg dFz2 receptor allow measurements of the receptor at the NMJ synapse (dFz2N; Figure S9) and the cleaved fragment (dFz2C; Figure 5) imported into muscle nuclei [57], [59]. We first assayed dFz2 receptor abundance at the NMJ with the N-terminal specific antibody. The dFz2 receptor is closely associated with the synaptic cell membrane marker FasII and occupies a domain that envelopes all type Ib boutons (Figure S9A). In *hs6st* nulls, the dFz2 receptor domain was spatially extended as compared to controls, however *sulf1* alleles showed no detectable change in the receptor. Likewise, fluorescence intensity measurements showed no significant difference between control and *sulf1* nulls, but *hs6st* null synapses displayed a ∼25% increase in dFz2 receptor abundance, a very significant elevation (p<0.01, n = 12; Figure S9B) in synaptic dFz2 abundance. Thus, importantly (see Discussion), significantly more dFz2 receptors occur in the *hs6st* null compared to *sulf1* null synapse.

To assay downstream signal transduction, the cleaved Fz2C fragment imported into muscle nuclei was quantified using the established method of counting dFz2C-positive punctae in nuclei proximal to the NMJ (Figure 5) [59]. In genetic control (*w^1118^*), most muscle nuclei contained a small number (1–3) of detectable dFz2C punctae, but some nuclei contained more and others were devoid of detectable dFz2C (Figure 5A, top). More than 100 muscle nuclei were quantified in >7 different animals to determine the control level of dFz2C nuclear import. In *sulf1* and *hs6st* mutants, there was a clear and consistent bidirectional difference in the number and size of dFz2C punctae in muscle nuclei (Figure 5A, middle and bottom). Null *sulf1* nuclei showed a highly significant decrease in number of dFz2C punctae per nuclei (>50% decreased; p<0.01; n = 163; Figure 5B). In contrast, *hs6st* nulls had an opposing highly significant increase in dFz2C punctae per nuclei (>60% increased; p<0.01; n = 163; Figure 5B). The difference between *sulf1* and *hs6st* null mutants was very highly significant (p<0.001), with a differential change in signaling paralleling the bidirectional change in synaptic functional differentiation (Figure 2).

**Figure 6:**
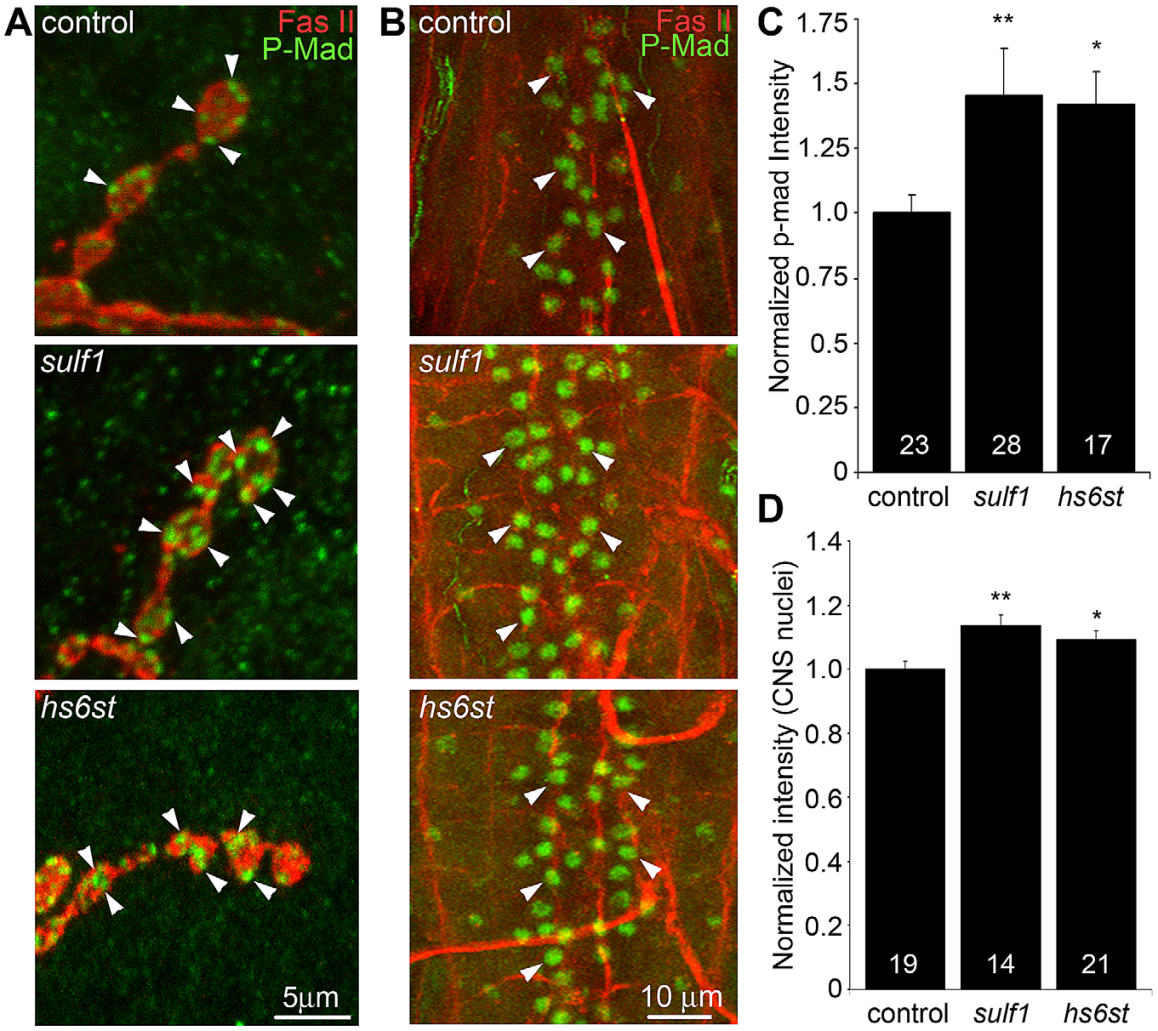
Loss of *sulf1* and *hs6st* causes differential effects on BMP signaling. (A) Representative NMJ synaptic boutons on muscle 6 in segment A3 from control (*w^1118^*), *sulf1* and *hs6st* nulls, labeled with neural marker anti-Fasciclin II (FasII, red) and for phosphorylated Mothers against decapentaplegic (P-Mad; green) activated downstream of Gbb signaling. Arrows indicate representative P-Mad punctae in the indicated genotypes. (B) Representative ventral nerve cord (VNC) midlines from the same 3 genotypes, labeled with anti-FasII (red) and P-Mad (green). Labeled motor neuron nuclei are indicated by arrows. Quantification of the mean fluorescent intensity level of P-Mad labeling normalized to FasII co-label at the NMJ synapse (C) and in motor neuron nuclei (D), normalized to genetic control. Sample sizes are ≥14 animals per indicated genotypes. Statistically significant differences calculated using the Mann-Whitney test for non-parametric data, * p<0.05, ** p<0.01. Error bars indicate S.E.M.

A characterized antibody specifically recognizing phosphorylated Mad (P-Mad) allowed independent measurements of Gbb signaling in the presynaptic terminal and P-Mad import into the motor neuron nuclei as a transcriptional regulator (Figure 6) [25], [60]. To assay this transduction pathway, P-Mad fluorescent intensity normalized to FasII was first assayed in presynaptic boutons [61], [62]. In the genetic control (*w^1118^*), P-Mad labeling was bounded by the synaptic cell adhesion molecule marker FasII, with P-Mad localized in numerous punctate domains (Figure 6A, arrows). In *sulf1* and *hs6st* nulls, both the intensity and size of P-Mad positive punctae were obviously and consistently greater than in controls (Figure 6A, middle and bottom). In fluorescence intensity quantification, *sulf1* null synapses displayed a significant increase in synaptic P-Mad (45% increased; p<0.05; n = 10; Figure 6C). An increase in P-Mad was also observed in the *hs6st* null boutons (42% greater than control; p<0.01; n = 15; Figure 6C). The motor neuron nuclei at the ventral nerve cord (VNC) midline accumulate P-Mad transcription factor downstream of Gbb signaling at the NMJ [25], [61], [62]. In genetic control (*w^1118^*), P-Mad nuclear labeling was consistently detected in these motor neuron nuclei (Figure 6B, arrows). A similar P-Mad distribution was observed in motor neuron nuclei of *sulf1* and *hs6st* nulls, but the intensity of P-mad expression was clearly and consistently elevated in both mutants compared to control (Figure 6B, middle and bottom). In fluorescence intensity quantification, *sulf1* null neuronal nuclei displayed a very significant increase in P-Mad accumulation (15% increased; p<0.01; n = 14; Figure 6D), paralleling increased P-Mad signaling at the NMJ (Figure 6C). Likewise, *hs6st* null motoneuron nuclei exhibited a smaller but still significant elevation in P-Mad accumulation (9% elevated over control; p<0.05; n = 21; Figure 6D), again paralleling the observed P-Mad signaling change at the NMJ (Figure 6C). We conclude that both anterograde WNT (Wg) and retrograde BMP (Gbb) *trans*-synaptic signaling in muscle and motor neuron nuclei, respectively, is differentially regulated by the *sulf1* and *hs6st* HSPG sulfation mechanism.

### *Trans*-synaptic WNT/BMP signals genetically interact with *sulf1* and *hs6st* nulls

**Figure 7:**
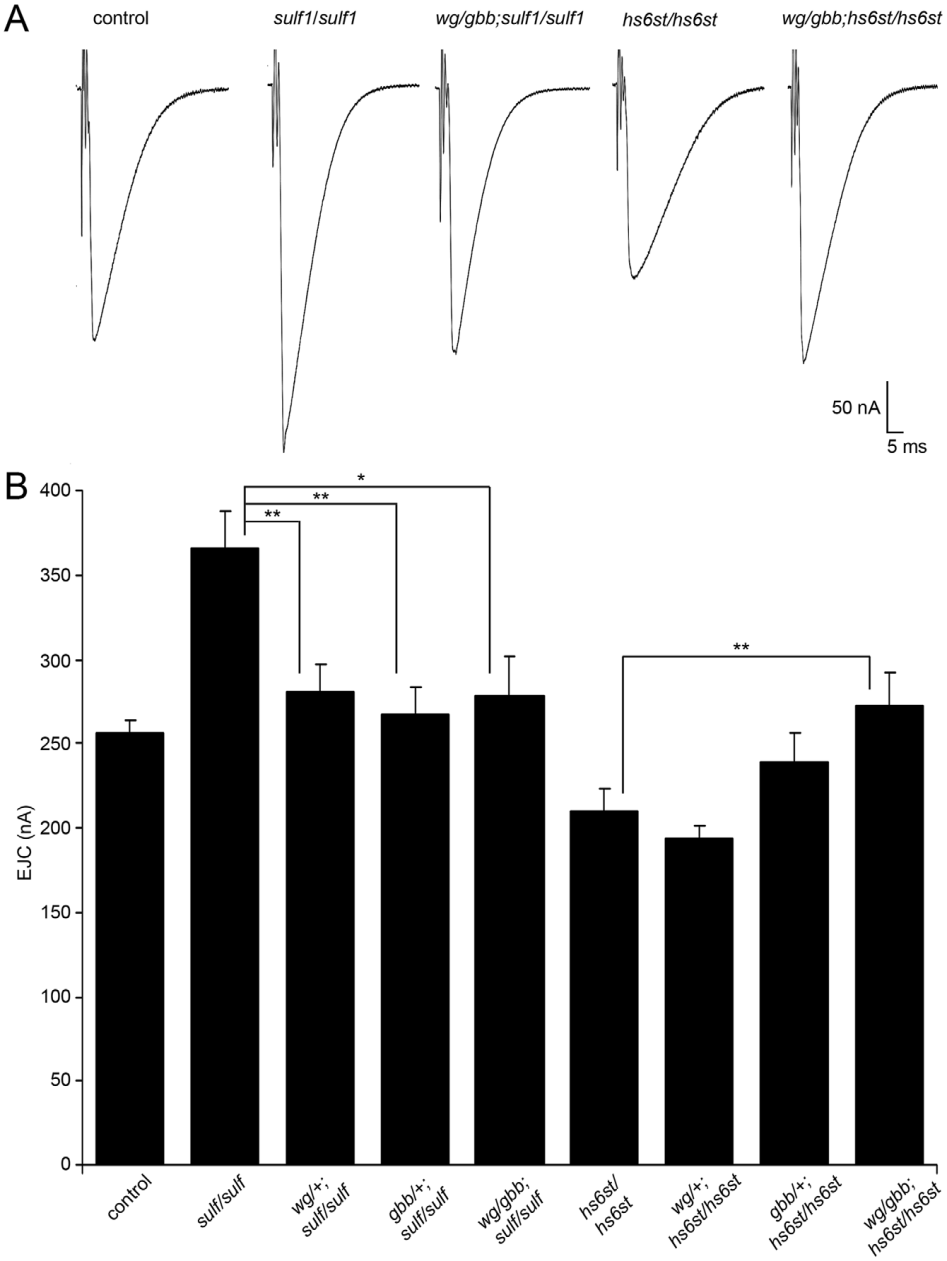
WNT and BMP signals genetically interact with *sulf1* and *hs6st* nulls. Genetic reduction of Wg and Gbb levels in *sulf1* and *hs6st* homozygous conditions restore EJC amplitudes to control levels. (A) Representative excitatory junctional current (EJC) traces from control (*w^1118^*), homozygous *sulf1^Δ1^* null, heterozygous *wg*/+ and *gbb*/+ in *sulf1* null background (*wg^I-12^/gbb^2^*; *sulf1^Δ1^*/*sulf1^Δ1^*), homozygous *hs6st^d770^* null and heterozygous *wg*/+ and *gbb*/+ in *hs6st* null background (*wg^I-12^/gbb^2^*; *hs6st^d770^*/*hs6st^d770^*). The nerve was stimulated (arrows) in 1.0 mM external Ca^2+^, and TEVC records (−60 mV holding potential) made from muscle 6 in segment A3. Each trace was averaged from 10 consecutive evoked EJC recordings. (B) Quantified mean EJC amplitudes (nA) for the nine genotypes shown. Sample sizes are ≥7 animals per indicated genotype. Statistically significant differences calculated using student's t-test, * p<0.05, ** p<0.01. Error bars indicate S.E.M.

In the *sulf1* and *hs6st* nulls we identified a bi-directional change in synaptic functional differentiation, measured as evoked junction current amplitudes increased in *sulf1* and decreased in *hs6st* null synapses (Figure 2). We therefore hypothesized that these functional changes are driven by the differential Wg and Gbb *trans*-synaptic signaling defects characterized above in *sulf1* and *hs6st* mutants (Figure 3, Figure 4, Figure 5, Figure 6). We reasoned that correcting Wg and Gbb levels in *sulf1* and *hs6st* nulls should restore neurotransmission to control levels. To test this hypothesis, we crossed heterozygous *wg*/+ and *gbb*/+ mutants into both *sulf1* and *hs6st* homozygous null backgrounds, both singly and in combination, and compared them to both positive and negative controls. The resulting 9 genotypes were all assayed with TEVC electrophysiology to compare EJC transmission strength. A summary of these data is given in Figure 7.

Representative transmission records are shown as an average of 10 consecutive EJC responses (1.0 mM extracellular Ca^2+^) for the genotypes in Figure 7A, with quantification of mean peak amplitudes in all genotypes shown in Figure 7B. First testing *sulf1* nulls, we examined the consequences of heterozygous genetic reduction of Wg and Gbb, alone and in combination. Compared to the elevated EJC amplitude of the *sulf1* null condition (381.28±1 62.24 nA, p<0.01, n = 9; Figure 7B), genetic reduction of Wg (*wg/+; sulf1/sulf1*) caused very significantly reduced transmission, similar to genetic reduction of Gbb (*gbb/+; sulf1/sulf1*) with a comparable effect, restoring EJC amplitude to control levels (267.16±16.33, p<0.01, n = 9; Figure 7B). Combinatorial genetic reduction of both Wg and Gbb in the *sulf1* null (*wg/gbb;sulf1/sulf1*) similarly returned EJC amplitudes to control levels (278.78±23.17, n = 7; Figure 7B). Secondly testing *hs6st* nulls, genetic reduction of either Wg or Gbb alone was not sufficient to significantly change the depressed synaptic function (Figure 7B). In this case, combinatorial genetic reduction of both Wg and Gbb in the *hs6st* null (*wg/gbb;hs6st/hs6st*) was required to raise the depressed EJC amplitude, a very significant increase back to control levels (272.98±18.58, p<0.01, n = 8; Figure 7B). Therefore, we conclude that combinatorial Wg and Gbb *trans*-synaptic signaling defects are causative for the observed bi-directional effects on synaptic functional differentiation in the *sulf1* and *hs6st* null mutant conditions.

### The *sulf1* and *hs6st* mechanism regulates pre- and postsynaptic differentiation

**Figure 8:**
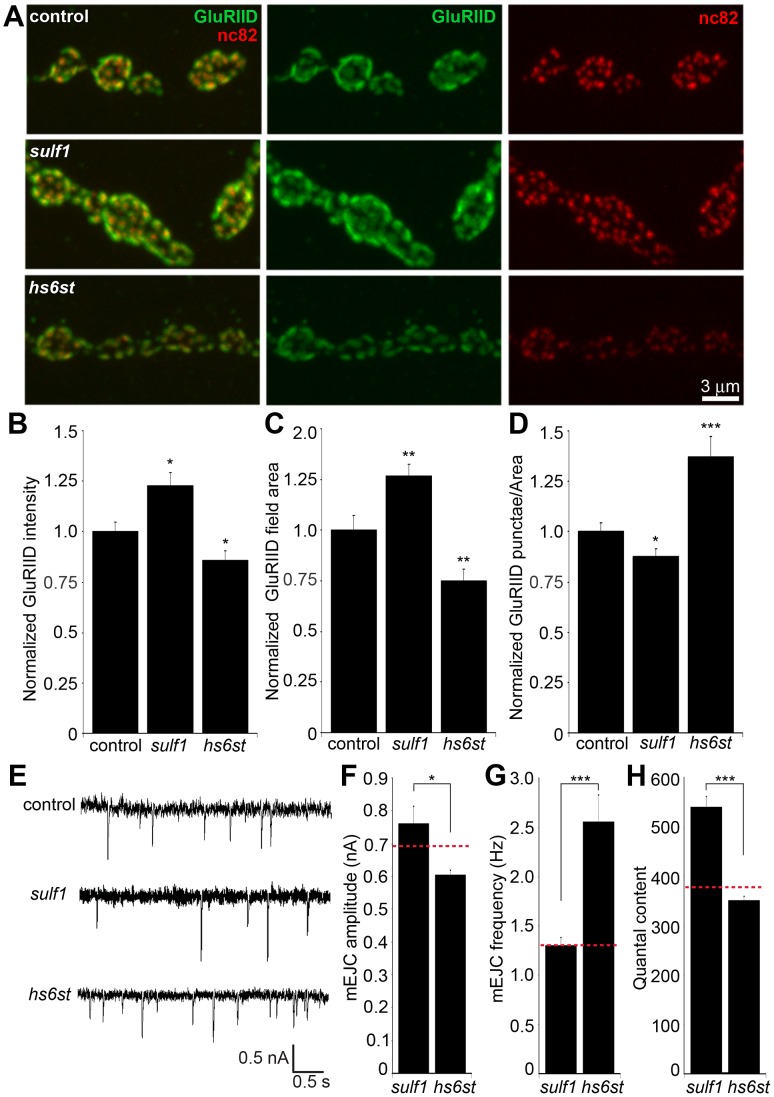
Bi-directional effects of *sulf1* and *hs6st* nulls on synaptic assembly. (A) Representative NMJ boutons from control (*w^1118^*), *sulf1* and *hs6st* null genotypes, labeled for postsynaptic Bad Reception (Brec) glutamate receptor IID subunit (GluRIID, green) and presynaptic active zone Bruchpilot (anti-nc82, red). Quantification of GluRIID mean fluorescent intensity (≥18 animals per indicated genotype) (B), GluRIID field area (≥40 boutons from ≥9 animals per indicated genotype) (C), and GluRIID punctae number per synaptic bouton (≥40 boutons from ≥9 animals per indicated genotype) (D), all normalized to genetic control. (E) Representative mEJC traces from control (*w^1118^*), *sulf1^Δ1^* and *hs6st^d770^* null alleles. Quantified mean mEJC amplitude (nA) (F), mean mEJC frequency (Hz) (G) and mean quantal content (H), with genetic control levels indicated as a dotted red line in each case. Sample sizes ≥15 recordings per indicated genotype. Statistically significant differences calculated using student's t-test or Mann-Whitney test for non-parametric data and indicated as, * p<0.05, ** p<0.01, *** p<0.001. Error bars indicate S.E.M.

The consequence of WNT (Wg) and BMP (Gbb) *trans*-synaptic signaling is nuclear import and transcriptional regulation in both synaptic partner cells [49], [51]. We therefore hypothesized that *sulf1* and *hs6st* null mutants would show bidirectional changes in pre- and postsynaptic molecular components that would explain the bidirectional change in synaptic functional differentiation (Figure 2 and Figure 7). To test this hypothesis, we examined a key component of the presynaptic active zone (Bruchpilot; Brp) [63], and an essential subunit of the postsynaptic glutamate receptor (Bad Reception (Brec); GluRIID) [64]. In parallel, we also performed a miniature EJC (mEJC) analysis to compare functional presynaptic vesicle release probability and postsynaptic response amplitude. A summary of these data is shown in Figure 8.

First, NMJ synapses were double-labeled for GluRIID recognized with anti-Brec (green) and Brp recognized with anti-nc82 (red) to compare genetic control (*w^1118^*) with *sulf1* and *hs6st* nulls (Figure 8A). We found that GluRIID was very significantly elevated at *sulf1* synapses compared to control (∼30% increased; p<0.01, n = 20; Figure 8B). In the opposing direction, *hs6st* null synapses showed a significant decrease in GluRIID abundance (∼15% reduced; p<0.05, n = 21; Figure 8B). The GluRIID field area per bouton and number of GluRIID punctae normalized to field area per synaptic bouton were also bidirectionally altered in the *sulf1* and *hs6st* nulls (Figure 8C, 8D). GluRIID receptor field area was increased in *sulf1* (∼30% greater; p<0.01, n = 47) but decreased in *hs6st* (∼25% reduced; p<0.01, n = 51). Conversely, measurements of GluRIID puncta normalized to field area per synaptic bouton were decreased in *sulf1* (∼15% lower; p<0.05, n = 47), but increased in *hs6st* nulls (∼40% greater; p<0.01, n = 51, Figure 8D). The bi-directional differences between *sulf1* and *hs6st* were very highly significant (p<0.001). The active zone protein Brp also showed opposite effects (Figure 8A). Although the difference between *sulf1* null and control was not quite significant (p>0.05, n = 20), *hs6st* null synapses showed a very significant decrease in Brp compared to control (∼20% reduced; p<0.01, n = 21; Figure 8A).

Based on these results, we next tested pre- (Brp) and postsynaptic (Brec/GluRIID) changes in *sulf1* and *hs6st* mutants with genetic reduction of Wg and Gbb (*wg/gbb;sulf1/sulf1* and *wg/gbb;hs6st/hs6st*), as in Figure 7. Distribution changes of both pre- and postsynaptic components were assayed as measurements of glutamate receptor field and active zone areas (Figure S10A). To measure glutamate receptor distribution comparing *wg/gbb;sulf1/sulf1* to matched control, we counted the number of GluRIID punctae per bouton (p = 0.73, n = 48; Figure S10B) and GluRIID area (p = 0.92, n = 48; Figure S10C), and found both corrected back to control levels. Likewise, for *wg/gbb;hs6st/hs6st* compared to control, GluRIID puncta number (p = 0.88, n = 48) and area (p = 0.41, n = 58) were both corrected to control levels. To measure Brp-positive presynaptic active zones comparing *wg/gbb;sulf1/sulf1* to matched control, we counted the number of Brp punctae per bouton (p = 0.43, n = 48; Figure S10D) and Brp area (p = 0.39, n = 48; Figure S10D), and found both corrected back to control levels. Likewise, for *wg/gbb;hs6st/hs6st* compared to control, Brp number (p = 0.54, n = 58) and area (p = 0.19, n = 58) were also corrected back to control levels. These results provide strong genetic evidence that Wg and Gbb *trans*-synaptic signaling changes are causative for the pre- and postsynaptic molecular differentiation defects in the *sulf1* and *hs6st* null mutants.

These bidirectional pre- and postsynaptic molecular changes parallel functional transmission changes in *sulf1* and *hs6st* mutants (Figure 2). To assay function at the single synapse level, we finally assayed spontaneous synaptic vesicle fusion events. Representative mEJC traces for control compared to *sulf1* and *hs6st* nulls are shown in Figure 8E. Consistent with observed bidirectional changes in evoked transmission, mEJC amplitudes in *hs6st* were ∼25% lower than in *sulf1* nulls (*hs6st,* 0.60±0.02 nA vs. *sulf1*, 0.76±0.05 nA; p<0.5, n = 34; Figure 8F). Moreover, *hs6st* nulls had a ∼100% elevated mEJC frequency compared to *sulf1* nulls (*hs6st*, 2.56±0.27 vs. *sulf1,* 1.30±0.09; p<0.001, n = 34; Figure 8G). Based on these mEJC measurements, there was a highly significant bidirectional change in quantal content between the two mutant conditions, with *sulf1* quantal content ∼50% greater than *hs6st* (*sulf1,* 539.98±22.02 vs. *hs6st*, 350.69±8.92; p<0.001, n = 34; Figure 8H). Taken together, these results show a bi-directional change in presynaptic glutamate release machinery and vesicle fusion probability, as well as postsynaptic glutamate receptor levels and functional responsiveness. We conclude that these changes underlie the bi-directional switch in neurotransmission strength characterizing *sulf1* and *hs6st* mutants.

## Discussion

It is well known that synaptic interfaces harbor heavily-glycosylated membrane proteins, glycolipids and ECM molecules, but understanding of glycan-mediated mechanisms within this synaptomatrix is limited [9]. Our genomic screen aimed to systematically interrogate glycan roles in both structural and functional development in the genetically-tractable *Drosophila* NMJ synapse. 130 candidate genes were screened, classified into 8 functional families: N-glycan biosynthesis, O-glycan biosynthesis, GAG biosynthesis, glycoprotein/proteoglycan core proteins, glycan modifying/degrading enzymes, glycosyltransferases, sugar transporters and glycan-binding lectins. From this screen, 103 RNAi knockdown conditions were larval viable, whereas 27 others produced early developmental lethality. 35 genes had statistically significant effects on different measures of morphological development: 27 RNAi-mediated knockdowns increased synaptic bouton number, 9 affected synapse area (2 increased, 7 decreased) and 2 genes increased synaptic branch number. These data suggest that overall glycan mechanisms predominantly serve to limit synaptic morphogenesis. 13 genes had significant effects on the functional differentiation of the synapse, with 12 increasing transmission strength and only 1 decreasing function upon RNAi knockdown. Thus, glycan-mediated mechanisms also predominantly limit synaptic functional development. A very small fraction of tested genes (CG1597; *pgant35A*, CG7480; *veg*, CG6657; *hs6st*, CG4451; *sulf1,* CG6725 and CG11874) had effects on both morphology and function. A large percentage of genes (∼30%) showed morphological defects with no corresponding effect on function, while only 7% of genes showed functional alterations without morphological defects, and <5% of all genes affect both. These results suggest that glycans have clearly separable roles in modulating morphological and functional development of the NMJ synapse.

A growing list of neurological disorders linked to the synapse are attributed to dysfunctional glycan mechanisms, including muscular dystrophies, cognitive impairment and autism spectrum disorders [65], [66], [67]. *Drosophila* homologs of glycosylation genes implicated in neural disease states include *ALG3* (CG4084), *ALG6* (CG5091), *DPM1* (CG10166), *FUCT1* (CG9620), *GCS1* (CG1597), *MGAT2* (CG7921), *MPDU1* (CG3792), *PMI* (CG33718) and *PPM2* (CG12151) [65]. Two of these genes, *Gfr* (CG9620) and CG1597, showed synaptic morphology phenotypes in our RNAi screen. Given that connectivity defects are clearly implicated in cognitive impairment and autism spectrum disorders [68], [69], it would be of interest to explore the glycan mechanism affecting synapse morphology in *Drosophila* models of these disease states. Glycans are well known to modulate extracellular signaling, including ligands of integrin receptors, to regulate intercellular communication [70], [71]. In our genetic screen, several O-glycosyltransferases mediating this mechanism were identified to show morphological (*GalNAc-T2*, CG6394; *pgant35A,* CG7480, *O-fut2,* CG14789; *rumi,* CG31152) and functional (*pgant5,* CG31651; *pgant35A,* CG7480) synaptic defects upon RNAi knockdown. These findings suggest that known integrin-mediated signaling pathways controlling NMJ synaptic structural and functional development [16], [41], [72], [73] are modulated by glycan mechanisms. Our screen showed CG6657 RNAi knockdown affects functional differentiation, consistent with reports that this gene regulates peripheral nervous system development [74]. The corroboration of our screen results with published reports underscores the utility of RNAi-mediated screening to identify glycan mechanisms, and supports use of our screen results for bioinformatic/meta-analysis to link observed phenotypes to neurophysiological/pathological disease states and to direct future glycan mechanism studies at the synapse.

From our screen, the two functionally-paired genes *sulf1* and *hs6st* were selected for further characterization. As in the RNAi screen, null alleles of these two genes had opposite effects on synaptic functional differentiation but similar effects on synapse morphogenesis, validating the corresponding screen results. The two gene products have functionally-paired roles; Hs6st is a heparan sulfate (HS) 6-O-sulfotransferase [43], and Sulf1 is a HS 6-O-endosulfatase [75]. These activities control sulfation of the same C_6_ on the repeated glucosamine moiety in HS GAG chains found on heparan sulfate proteoglycans (HSPGs). At the *Drosophila* NMJ, two HSPGs are known to regulate synapse assembly; the GPI-anchored glypican Dally-like protein (Dlp), and the transmembrane Syndecan (Sdc) [45]. In contrast, the secreted HSPG Perlecan (Trol) is not detectably enriched at the NMJ [76], and indeed appears to be selectively excluded from the perisynaptic domain. In other developmental contexts, the membrane HSPGs Dlp and Sdc are known to act as co-receptors for WNT and BMP ligands, regulating ligand abundance, presentation to cognate receptors and therefore signaling [20], [48]. Importantly, the regulation of HSPG co-receptor abundance has been shown to be dependent on sulfation state mediated by extracellular sulfatases [77]. Consistently, we observed upregulation of Dlp and Sdc in *sulf1* null synapses, whereas Dlp was reduced in *hs6st* null synapses. In the developing *Drosophila* wing disc, HSPG co-receptors increase levels of the Wg ligand due to extracellular stabilization [78], and the primary function of Dlp in this developmental context is to retain Wg at the cell surface [21]. Likewise, in developing *Drosophila* embryos, a significant fraction of Wg ligand is retained on the cell surfaces in a HSPG-dependent manner [79], with the HSPG acting as an extracellular co-receptor. Syndecan also modulates ligand-dependent activation of cell-surface receptors by acting as a co-receptor [19], [20]. At the NMJ, regulation of both these HSPG co-receptors occurs in the closely juxtaposed region between presynaptic bouton and muscle subsynaptic reticulum, in the exact same extracellular space traversed by the secreted *trans*-synaptic Wg and Gbb signals [45]. We therefore proposed that altered Dlp and Sdc HSPG co-receptors in *sulf1* and *hs6st* mutants differentially trap/stabilize Wg and Gbb *trans*-synaptic signals at the interface between motor neuron and muscle, to modulate the extent and efficacy of intercellular signaling driving synaptic development.

HS sulfation modification is linked to modulating the intercellular signaling driving neuronal differentiation [80]. In particular, WNT and BMP ligands are both regulated via HS sulfation of their extracellular co-receptors, and both signals have multiple functions directing neuronal differentiation, including synaptogenesis [49], [50], [51]. In the *Drosophila* wing disc, extracellular WNT (Wg) ligand abundance and distribution was recently shown to be strongly elevated in *sulf1* null mutants [22]. Moreover, *sulf1* has also recently been shown to modulate BMP signaling in other cellular contexts [81]. Consistently, we have shown here increased WNT Wg and the BMP Gbb abundance and distribution in *sulf1* null NMJ synapses. The *hs6st* null also exhibits elevated Wg and Gbb at the synaptic interface, albeit the increase is lower and results in differential signaling consequences. In support of this contrasting effect, extracellular signaling ligands are known to bind HSPG HS chains differentially dependent on specific sulfation patterns [82], [83], [84]. It is important to note that the *sulf1* and *hs6st* modulation of *trans*-synaptic signals is not universal, as Jelly Belly (Jeb) ligand abundance and distribution was not altered in the *sulf1* and *hs6st* null conditions [17]. This indicates that discrete classes of secreted *trans*-synaptic molecules are modulated by distinct glycan mechanisms to control NMJ structure and function.

At the *Drosophila* NMJ, Wg is very well characterized as an anterograde *trans*-synaptic signal [23], [24], [85] and Gbb is very well characterized as a retrograde *trans*-synaptic signal [25], [26], [50], [86]. In Wg signaling, the dFz2 receptor is internalized upon Wg binding and then cleaved so that the dFz2-C fragment is imported into muscle nuclei [57], [59], [85]. In *hs6st* nulls, increased Wg ligand abundance at the synaptic terminal corresponds to an increase in dFz2C punctae in muscle nuclei as expected. In contrast, the increase in Wg at the *sulf1* null synapse did not correspond to an increase in the dFz2C-terminus nuclear internalization, but rather a significant decrease. One explanation for this apparent discrepancy is the ‘exchange factor’ model based on the biphasic ability of the HSPG co-receptor Dlp to modulate Wg signaling [48]. In the *Drosophila* wing disc, this model suggests that the transition of Dlp co-receptor from an activator to repressor of signaling depends on Wg cognate receptor dFz2 levels, such that a low ratio of Dlp∶dFz2 potentiates Wg-dFz2 interaction, whereas a high ratio of Dlp∶dFz2 prevents dFz2 from capturing Wg [48]. In *sulf1* null synapses, we observe a very great increase in Dlp abundance (∼40% elevated) with no significant change in the dFz2 receptor. In contrast, at *hs6st* null synapses there is a decrease in Dlp abundance (15% decreased) together with a significant increase in dFz2 receptor abundance (∼25% elevated). Thus, the higher Dlp∶dFz2 ratio in *sulf1* nulls could explain the decrease in Wg signal activation, evidenced by decreased dFz2-C terminus import into the muscle nucleus. In contrast, the Dlp∶Fz2 ratio in *hs6st* is much lower, supporting activation of the dFz2-C terminus nuclear internalization pathway. This previously proposed competitive binding mechanism dependent on Dlp co-receptor and dFz2 receptor ratios predicts the observed synaptic Wg signaling pathway modulation in *sulf1* and *hs6st* dependent manner [48].

At the *Drosophila* NMJ, Gbb is very well characterized as a retrograde *trans*-synaptic signal, with muscle-derived Gbb causing the receptor complex Wishful thinking (Wit), Thickveins (Tkv) and Saxaphone (Sax) to induce phosphorylation of the transcription factor mothers against Mothers against decapentaplegic (P-Mad) [25], [26], [87]. Mutation of Gbb ligand, receptors or regulators of this pathway have shown that Gbb-mediated retrograde signaling is required for proper synaptic differentiation and functional development [25], [52], [61], [86], [88]. Further, loss of Gbb signaling results in significantly decreased levels of P-Mad in the motor neurons [25]. We show here that accumulation of Gbb in *sulf1* and *hs6st* null synapses causes elevated P-Mad signaling at the synapse and P-Mad accumulation in motor neuron nuclei. Importantly, *sulf1* null synapses show a significantly higher level of P-Mad signaling compared to *hs6st* null synapses, and this same change is proportionally found in P-Mad accumulation within the motor neuron nuclei. These findings indicate differential activation of Gbb *trans*-synaptic signaling dependent on the HS sulfation state is controlled by the *sulf1* and *hs6st* mechanism, similar to the differential effect observed on Wg *trans*-synaptic signaling. Our genetic interaction studies show that these differential effects on *trans*-synaptic signaling have functional consequences, and exert a causative action on the observed bi-directional functional differentiation phenotypes in *sulf1* and *hs6st* nulls. Genetic correction of Wg and Gbb defects in the *sulf1* null background restores elevated transmission back to control levels. Similarly, genetic correction of Wg and Gbb in *hs6st* nulls restores the decreased transmission strength back to control levels. These results demonstrate that the Wg and Gbb *trans-*synaptic signaling pathways are differentially regulated and, in combination, induce opposite effects on synaptic differentiation.

Both *wg* and *gbb* pathway mutants display disorganized and mislocalized presynaptic components at the active zone (e.g. Bruchpilot; Brp) and postsynaptic components including glutamate receptors (e.g. Bad reception; Brec/GluRIID) [23], [86], [89]. Consistently, the bi-directional effects on neurotransmission strength in *sulf1* and *hs6st* mutants are paralleled by dysregulation of these same synaptic components. Changes in presynaptic Brp and postsynaptic GluR abundance/distribution causally explain the bi-directional effects on synaptic functional strength between *sulf1* and *hs6st* null mutant states. Alterations in active zone Brp and postsynaptic GluRs also agree with assessment of spontaneous synaptic activity. Null *sulf1* and *hs6st* synapses showed opposite effects on miniature evoked junctional current (mEJC) frequency (presynaptic component) and amplitude (postsynaptic component). Further, quantal content measurements also support the observation of bidirectional synaptic function in the two functionally paired nulls. Genetic correction of Wg and Gbb defects in both *sulf1* and *hs6st* nulls restores the molecular composition of the pre- and postsynaptic compartments back to wildtype levels. When both *trans-*synaptic signaling pathways are considered together, these data suggest that HSPG sulfate modification under the control of functionally-paired *sulf1* and *hs6st* jointly regulates both WNT and BMP *trans*-synaptic signaling pathways in a differential manner to modulate synaptic functional development on both sides of the cleft.

We present here the first systematic investigation of glycan roles in the modulation of synaptic structural and functional development. We have identified a host of glycan-related genes that are important for modulating neuromuscular synaptogenesis, and these genes are now available for future investigations, to determine mechanistic requirements at the synapse, and to explore links to neurological disorders. As proof for the utilization of these screen results, this study has identified extracellular heparan sulfate modification as a critical platform of the intersection for two secreted *trans*-synaptic signals, and differential control of their downstream signaling pathways that drive synaptic development. Other *trans*-synaptic signaling pathways are independent and unaffected by this mechanism, although it is of course possible that a larger assortment of signals could be modulated by this or similar mechanisms. This study supports the core hypothesis that the extracellular space of the synaptic interface, the heavily-glycosylated synaptomatrix, forms a domain where glycans coordinately mediate regulation of *trans*-synaptic pathways to modulate synaptogenesis and subsequent functional maturation.

## Materials and Methods

### *Drosophila* stocks and genetics

The glycan-related gene collection was generated using the KEGG glycan databases and Flybase annotation. The 163 UAS-RNAi lines tested were obtained from the Vienna *Drosophila* RNAi Center (VDRC) and Harvard TriP collection. Transgenic UAS-RNAi males were crossed to GAL4 driver females, with progeny raised at 25°C on standard food, controlling for density (3 ♀ crossed to 2 ♂). The UH1-GAL4 driver was used for ubiquitous knockdown of target gene expression [15]. Neural specific *elav*-GAL4 [90], muscle specific 24B-GAL4 [91] and glia specific *repo*-GAL4 lines [92] from Bloomington stock center were used to assay cell-targeted knockdown. The two *sulf1* null alleles used were *sulf1^Δ1^* [42] and *sulf1^ΔP1^* [43]. The two *hs6st* null alleles used were *hs6st^d770^* and the deficiency Df(3R)ED6027 [93]. The *wg* allele *wg^I-12^* [94] and *gbb* alleles *gbb^1^* and *gbb^2^* were used [25], [87]. Multiply mutant animals were made using standard genetic crosses. The trol-GFP line was obtained from Flytrap [76].

### Antibody production

We generated a rabbit polyclonal anti-Gbb antibody using a 1∶1 combination of two Gbb-specific peptides (SHHRSKRSASHP, NDENVNLKKYRNMIVKSC) corresponding to amino acids 319–330 and 435–452 of Gbb (Young-In Frontier, Seoul, Korea). The antibody was purified by Protein A affinity chromatography, and antibody specificity demonstrated by examining immunoreactivity in the wandering third instar neuromusculature with *gbb* mutants and by expressing *UAS-gbb^9.1^* under the control of the muscle driver BG57-GAL4 (Figure S11). Immunoreactivity in the wandering third instar neuromusculature was severely reduced in a strong hypomorphic *gbb* allele (*gbb^1^*/*gbb^2^*, *UAS-gbb^9.9^*), which has leaky expression of *UAS-gbb^9.9^* in a null allelic combination [25], [87], [95]. In sharp contrast, the anti-Gbb signal was strongly elevated in *BG57-GAL4/UAS-gbb^9.1^* relative to wildtype larvae.

### Immunocytochemistry

Wandering third instars were dissected in Ca^2+^-free saline and then immediately fixed in either 4% paraformaldehyde for 10 minutes (all labels except anti-Dlp) or Bouin's fixative for 30 mins (anti-Dlp). Preparations were then washed in permeabilizing PBST (PBS+0.1% Triton-X) or detergent-free PBS for extracellular labeling only [16]. The following primary antibodies were used: rabbit or goat anti-HRP (1∶250; Jackson ImmunoResearch Laboratories); mouse anti-DLG (4F3; 1∶250; Developmental Studies Hybridoma Bank (DSHB)); mouse anti–Fasciclin II (1D4; 1∶5; DSHB); mouse anti-Dlp (13G8, 1∶5; DSHB) and rabbit anti-Syndecan (1∶200) [96]; mouse anti-Wg (4D4; 1∶2 DSHB) and rabbit anti-Gbb (1∶100); rabbit anti-PcanV (1∶1000) [97]; guinea pig anti-Jeb (1∶100) [17]; rabbit anti-dFz2-C (1∶500) and rabbit anti-dFz2-N (1∶100) [57]; rabbit anti-Htl (1∶100) [56]; rabbit anti-P-Mad (PS1; 1∶1000) [60]; rabbit anti-GluRIID (1∶500) [64] and mouse anti-BRP (1∶100; DSHB). Primary antibodies were incubated at 4°C overnight. Alexa-conjugated secondary antibodies (Jackson ImmunoResearch Laboratories) were used at 1∶250 dilutions for 2 hours at room temperature. Staining with propidium iodide (Sigma Aldrich) to visualize cell nuclei was done at 1∶100 dilution of 1 mg/ml propidium iodide incubated for 30 minutes at room temperature.

### Imaging quantification

Images were taken with on an upright Zeiss LSM 510 META laser-scanning confocal using a Plan Apo 63× oil objective. For structural quantification, including NMJ synapse branch number, bouton number and area, preparations were double-labeled with anti-HRP and anti-DLG, with counts made at muscle 4 in segment A3. For nuclear import studies, nuclei were identified by propidium iodide staining with fluorescent punctae counted and intensity quantified [59]. For synaptic functional protein quantitation, glutamate receptor and Brp punctae were quantified for muscle 4, segment 3. Glutamate receptor number and field area was quantified in consecutive boutons of >3 µm diameter. All preparations were fixed, stained and processed simultaneously to allow for intensity comparisons. All analyses were done with ImageJ software (National Institutes of Health) using the threshold function to outline areas and Z-stacks made using the maximum projection function. Statistics were done either with one-way ANOVA analysis followed by Dunnett's post-test, student's t-test or Mann-Whitney test for non-parametric data. All analyses were done blind to genotypes during all stages of experimentation and analysis. All figure images were projected in LSM Image Examiner (Zeiss) and exported to Adobe photoshop.

### Heparin treatment

Stock solution of heparin (Sigma, H3393) in 1×PBS was prepared and serially diluted to obtain concentrations (e.g. 0.625, 0.315 and 0.156 mg/ml). Dissected wandering third instar larvae were incubated with these heparin concentrations for 5 minutes at RT, followed by a 1 minute wash with 1×PBS and then 10 minute fix with 4% paraformaldehyde in 1×PBS. After fixation, anti-Wg or anti-Gbb antibodies were used as above with appropriate secondary antibodies. Processed animals were analyzed for changes in intensity measurements as above in the image quantification section. All fluorescence intensity measurements were compared to preparations treated identically with only 1×PBS and no heparin, and the processed simultaneously for immunolabeling, microscopy and quantification.

### Electrophysiology

Two-electrode voltage-clamp (TEVC) records were made from the wandering third instar NMJ as previously described [41]. In brief, staged control, mutant and transgenic RNAi animals were secured on sylgard-coated coverslips with surgical glue (liquid suture), dissected longitudinally along the dorsal midline, and glued flat. The segmental nerves were cut near the base of the ventral nerve cord. Recording was performed in 128 mM NaCl, 2 mM KCl, 4 mM MgCl_2_, 1.0 mM CaCl_2_, 70 mM sucrose, and 5 mM Hepes. Recording electrodes (1-mm outer diameter capillaries; World Precision Instruments) were filled with 3 M KCl and had resistances of >15 MΩ. Spontaneous mEJCs were collected using continuous (gap-free) recording and evoked EJC recordings were made from the voltage-clamped (*V*_hold_ = −60 mV) muscle 6 in segment A3 with a TEVC amplifier (Axoclamp 200B; MDS Analytical Technologies). The cut segmental nerve was stimulated with a glass suction electrode at a suprathreshold voltage level (50% above baseline threshold value) for a duration of 0.5 ms. Records were made with 0.2 Hz nerve stimulation in episodic acquisition setting and analyzed with Clampex software (version 7.0; Axon Instruments). Each n = 1 represents a recording from a different animal. Statistical comparisons were performed using student's t-test or the Mann-Whitney test for non-parametric data.

## Supporting Information

Figure S1 NMJ synaptic bouton number in *sulf1* and *hs6st* mutants. (A) Representative NMJ images from muscle 4 in segment A3 showing anti-horseradish peroxidase (HRP; red) and anti-Discs Large (DLG; green) in control (*w^1118^*×UH1-GAL4), *sulf1* RNAi (UH1-GAL4×UAS-CG6725) and *hs6st* RNAi (UH1-GAL4×UAS-CG4451). (B) Quantification of synaptic bouton number in RNAi-knockdown conditions for *sulf1* and *hs6st*, normalized to genetic control (*w^1118^*×UH1-GAL4). Sample sizes are ≥10 animals per indicated genotypes. (C) Representative NMJ images of anti-HRP (red) and anti-DLG (green) in *w^1118^* control, *sulf1* and *hs6st* null mutants. (D) Quantification of synaptic bouton number in mutant conditions normalized to genetic control. Sample sizes are ≥8 animals per indicated genotype. Statistically significant differences were calculated using student's t-test and indicated as ***p<0.001. Error bars indicate S.E.M.
(TIF)

Figure S2 Double knockdown of *sulf1* and *hs6st* measure of EJC amplitude. (A) Representative evoked excitatory junctional current (EJC) traces from control (*w^1118^*×UH1-GAL-4) and double knockdown with both *sulf1* and *hs6st* RNAi transgenic lines (UH1-GAL4×UAS-*sulf1-*RNAi; UAS-*hs6st-*RNAi). (B) Quantified mean EJC amplitudes (nA) for the two genotypes shown in panel A normalized to control. Sample sizes are ≥12 animals per indicated genotype. Statistically significant differences calculated using student's t-test, * p<0.05, Error bars indicate S.E.M.
(TIF)

Figure S3 NMJ synaptic localization of Dally-like and Syndecan HSPGs. Representative confocal images showing HSPG synaptic localization at the larval NMJ. (A) Single channel images of presynaptic anti-horseradish peroxidase (anti-HRP, blue), Dally-like Protein (anti-Dlp, green) and postsynaptic glutamate receptor subunit IID (anti-GluRIID, red). (B) Single channel images showing presynaptic anti-horseradish peroxidase (anti-HRP, blue), syndecan (anti-Sdc, red) and postsynaptic Discs Large (anti-DLG, green). (C) Merged image showing Dlp localization with respect to presynaptic HRP, postsynaptic GluRIID and the triple-labeled terminal. (D) Merged image showing Sdc localization with respect to presynaptic HRP, postsynaptic DLG and the triple-labeled terminal.
(TIF)

Figure S4 HSPG Perlecan (Trol) is absent from the NMJ synaptic terminal. (A) Representative confocal image showing Perlecan expression at the wandering third instar larval NMJ using the Trol-GFP Flytrap line ZCL1700 from the Flytrap GFP Resource. Single channel and merged images show presynaptic anti-horseradish peroxidase (anti-HRP, red) and Trol-GFP (green). (B) Representative confocal image showing Perlecan (anti-PcanV) antibody staining, shown at a much higher confocal gain than in A to emphasize muscle expression. Perlecan is strongly expressed in the motor nerve, and clearly present on the muscle surface, but is never detectably enriched at the NMJ terminal. In many cases, as in the example shown, Perlecan appears at lower levels in the perisynaptic region surrounding the NMJ than elsewhere on the muscle.
(TIF)

Figure S5 Permeabilized versus non-permeabilized Wg and Gbb labeling. Representative NMJ images of muscle 6/7 in segment A3 from the wandering third instar. Merged and single channel images of (A) anti-horseradish peroxidase (HRP; red) and anti-Wingless (Wg; green), and (B) anti-Fasciclin II (FasII; green) and anti-glass bottom boat (Gbb; red), in non-permeablized labeling conditions in the absence of detergent. Note strong localization of both Wg and Gbb at the NMJ terminal. Merged and single channel images of (C) anti-HRP (red) and anti-Wg (green), and (D) anti-FasII (green) and anti-Gbb (red) in permeablized labeling conditions with 4% paraformaldehyde added to all antibody incubations. Note that most of the synaptic localization of Wg and Gbb is lost.
(TIF)

Figure S6 NMJ retention of Wg/Gbb altered by highly-sulfated heparin. Confocal imaging of Wg and Gbb *trans*-synaptic ligand abundance at the wandering third instar NMJ (muscle 4, segment A3) following acute incubation with highly-sulfated heparin. (A) Single channel and merged images of anti-horseradish peroxidase (HRP; red) and anti-Wingless (Wg; green) following control (no heparin), 0.156 mg/ml, 0.315 mg/ml and 0.625 mg/ml heparin treatments. (B) Single channel and merged images of anti-HRP (red) and anti-glass bottom boat (Gbb; green) following control, 0.156 mg/ml, 0.315 mg/ml and 0.625 mg/ml heparin treatments. (C) Quantification of fluorescence intensity of Wg and Gbb normalized to the internal HRP co-label for the control and indicated heparin concentrations. Individual data points are an average of ≥3 animals. Dotted line shows fitted linear trend lines. Statistically significant differences calculated using student's t-test and indicated as ***p<0.001, ** p<0.01, * p<0.05. Error bars indicate S.E.M.
(TIF)

Figure S7 NMJ expression of Jeb ligand unchanged in *sulf1*/*hs6st* nulls. (A) Representative NMJ images at the wandering third instar NMJ on muscle 6 in segment A3 from control (*w^1118^*), *sulf1* and *hs6st* nulls, labeled with neural marker anti-horseradish peroxidase (HRP; red) and anti-Jelly belly (Jeb; green). Merged images show Jeb tightly localized at synaptic boutons. (B) Quantification of anti-Jeb mean fluorescence intensity levels normalized to HRP co-label and the genetic control. Sample sizes are ≥8 animals per indicated genotypes. Statistically significant differences calculated using student's t-test. N.S. indicates no significant difference. Error bars indicate S.E.M.
(TIF)

Figure S8 NMJ expression of FGF receptor unchanged in *sulf1/hs6st* nulls. (A) Representative NMJ images at the wandering third instar NMJ on muscle 6 in segment A3 from control (*w^1118^*), *sulf1* and *hs6st* nulls, labeled with neural marker anti-horseradish peroxidase (HRP; red) and anti-Heartless (Htl; green). Merged images show the Htl FGF receptor tightly localized at synaptic boutons. (B) Quantification of Htl mean fluorescence intensity levels normalized to HRP co-label and the genetic control. Sample sizes are ≥7 animals per indicated genotypes. Statistically significant differences calculated using student's t-test. N.S. indicates no significant difference. Error bars indicate S.E.M.
(TIF)

Figure S9 Synaptic Frizzled-2 receptor levels in *sulf1* and *hs6st* nulls. Frizzled-2 receptor N-terminus (dFz2-N) specific antibody shows localized expression surrounding synaptic boutons at the NMJ. (A) Representative wandering third instar NMJ images from muscle 6 in segment A3 for control (*w^1118^*), *sulf1* and *hs6st* null mutants, double-labeled with presynaptic neural marker anti-Fasciclin II (FasII, red) and dFz2-N (green). Right: dFz2-N shown alone for clarity. (B) Quantification of dFz2-N mean fluorescence intensity for the indicated genotypes, normalized to the genetic control. Sample sizes are ≥12 animals per genotype. Statistically significant differences calculated using student's t-test, ** p<0.01. Error bars indicate S.E.M.
(TIF)

Figure S10 Wg and Gbb signals genetically interact with *sulf1* and *hs6st* nulls. Genetic reduction of Wg and Gbb levels in *sulf1* and *hs6st* homozygous conditions restores molecular synaptic assembly to control levels. (A) Representative NMJ boutons from control (*w^1118^*), heterozygous *wg*/+ and *gbb*/+ in *sulf1* null background (*wg^I-12^/gbb^2^*; *sulf1^Δ1^*/*sulf1^Δ1^*) and *hs6st* null background (*wg^I-12^/gbb^2^*; *hs6st^d770^*/*hs6st^d770^*) labeled for postsynaptic Bad Reception (Brec) glutamate receptor IID subunit (GluRIID, green) and presynaptic active zone Bruchpilot (anti-nc82, red). Quantification of GluRIID punctae/bouton (B), total GluRIID area (C), Brp punctae/bouton (D) and total Brp area (E), all normalized to the genetic control. All multiply mutant conditions are restored to control levels for all parameters, with no significant differences remaining.
(TIF)

Figure S11 Characterization of anti-Gbb antibody specificity. Representative confocal images of wandering third instar NMJ 6/7 double-labeled with anti-Gbb (red) and anti-HRP (green) under detergent permeabilized (A–C) and non-permeabilized (D–F) conditions. The genotypes analyzed include control (*w^1118^*; A,D), *gbb^1^*/*gbb^2^*,*UAS-gbb^9.9^* (B,E), and *BG57-GAL4/UAS-gbb^9.1^* (C,F).
(TIF)

Table S1 Primary screen results. Raw number values of the RNAi screen indicated by human ortholog name, *Drosophila* gene name and CG number. Mean value and standard deviation (SD) included for NMJ morphology parameters of bouton number, branch number and synaptic area, and for NMJ functional parameter of evoked excitatory junctional current (EJC) amplitude. Sample sizes ≥6 NMJs and ≥3 animals for morphology and function measurements.
(XLS)

Table S2 Secondary screen results. Raw number values for the secondary screen results indicated by human ortholog name, *Drosophila* gene name and CG number. The two independent IDs for RNAi lines are shown. For all retested lines, morphological quantification for NMJ bouton number (top) and evoked excitatory junctional current (EJC) amplitude (bottom). All results are shown as fold-changes compared to genetic control. Sample sizes are ≥6 individual animals per genotype. Replication of primary screen result is indicated in the final column as Y, and failure to replicate indicated as N.
(XLS)
