# Supplementary figures and images for: A Targeted Glycan-Related Gene Screen Reveals Heparan Sulfate Proteoglycan Sulfation Regulates WNT and BMP Trans-Synaptic Signaling
Source: PLoS Genet. 2012 Nov 8;8(11):e1003031. doi: 10.1371/journal.pgen.1003031 (PMC3493450; doi:10.1371/journal.pgen.1003031)

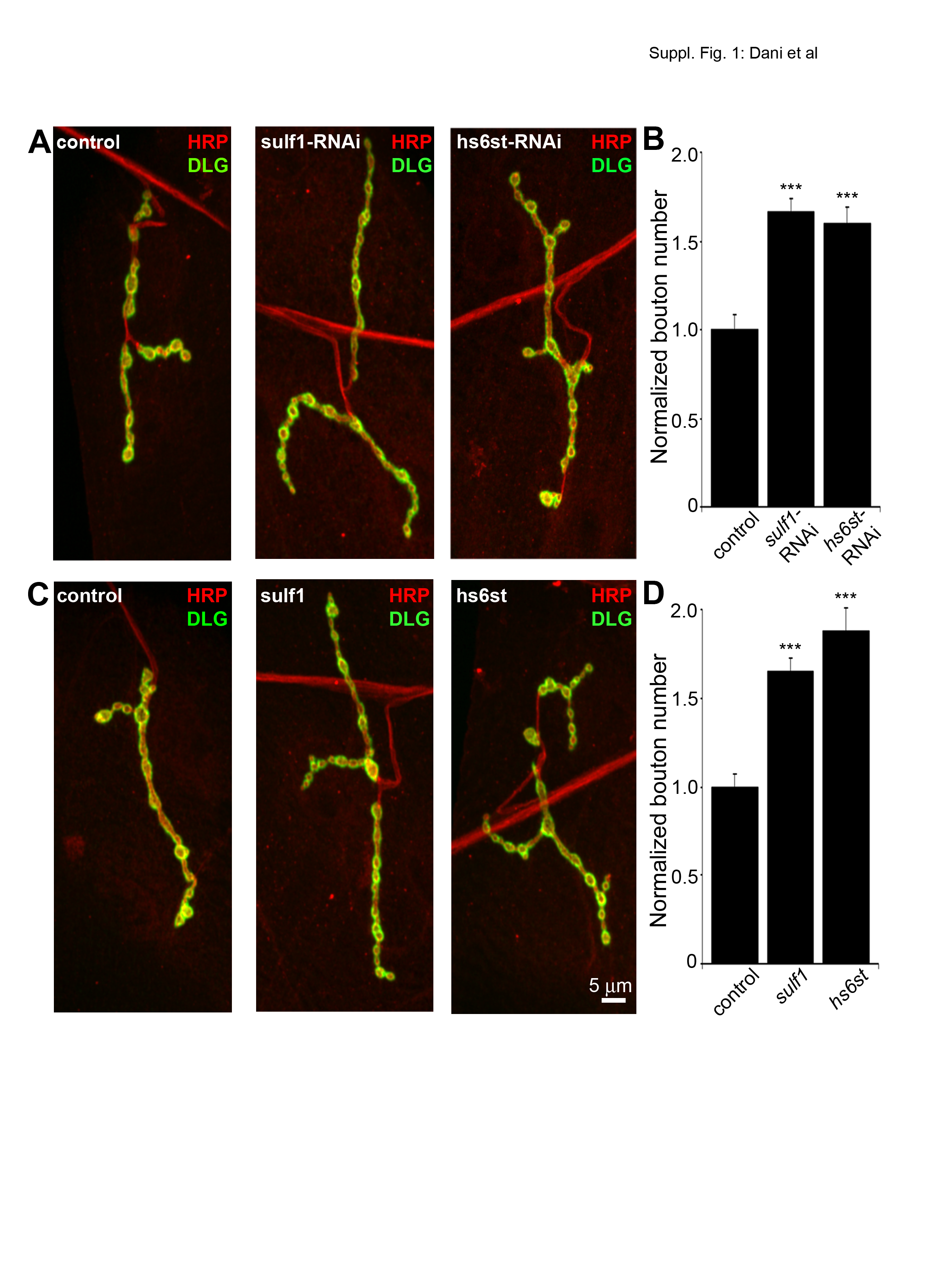

Supplement: Figure S1 — NMJ synaptic bouton number in sulf1 and hs6st mutants. (A) Representative NMJ images from muscle 4 in segment A3 showing anti-horseradish peroxidase (HRP; red) and anti-Discs Large (DLG; green) in control (w1118×UH1-GAL4), sulf1 RNAi (UH1-GAL4×UAS-CG6725) and hs6st RNAi (UH1-GAL4×UAS-CG4451). (B) Quantification of synaptic bouton number in RNAi-knockdown conditions for sulf1 and hs6st, normalized to genetic control (w1118×UH1-GAL4). Sample sizes are ≥10 animals per indicated genotypes. (C) Representative NMJ images of anti-HRP (red) and anti-DLG (green) in w1118 control, sulf1 and hs6st null mutants. (D) Quantification of synaptic bouton number in mutant conditions normalized to genetic control. Sample sizes are ≥8 animals per indicated genotype. Statistically significant differences were calculated using student's t-test and indicated as ***p<0.001. Error bars indicate S.E.M. (TIF) [file pgen.1003031.s001.tif]

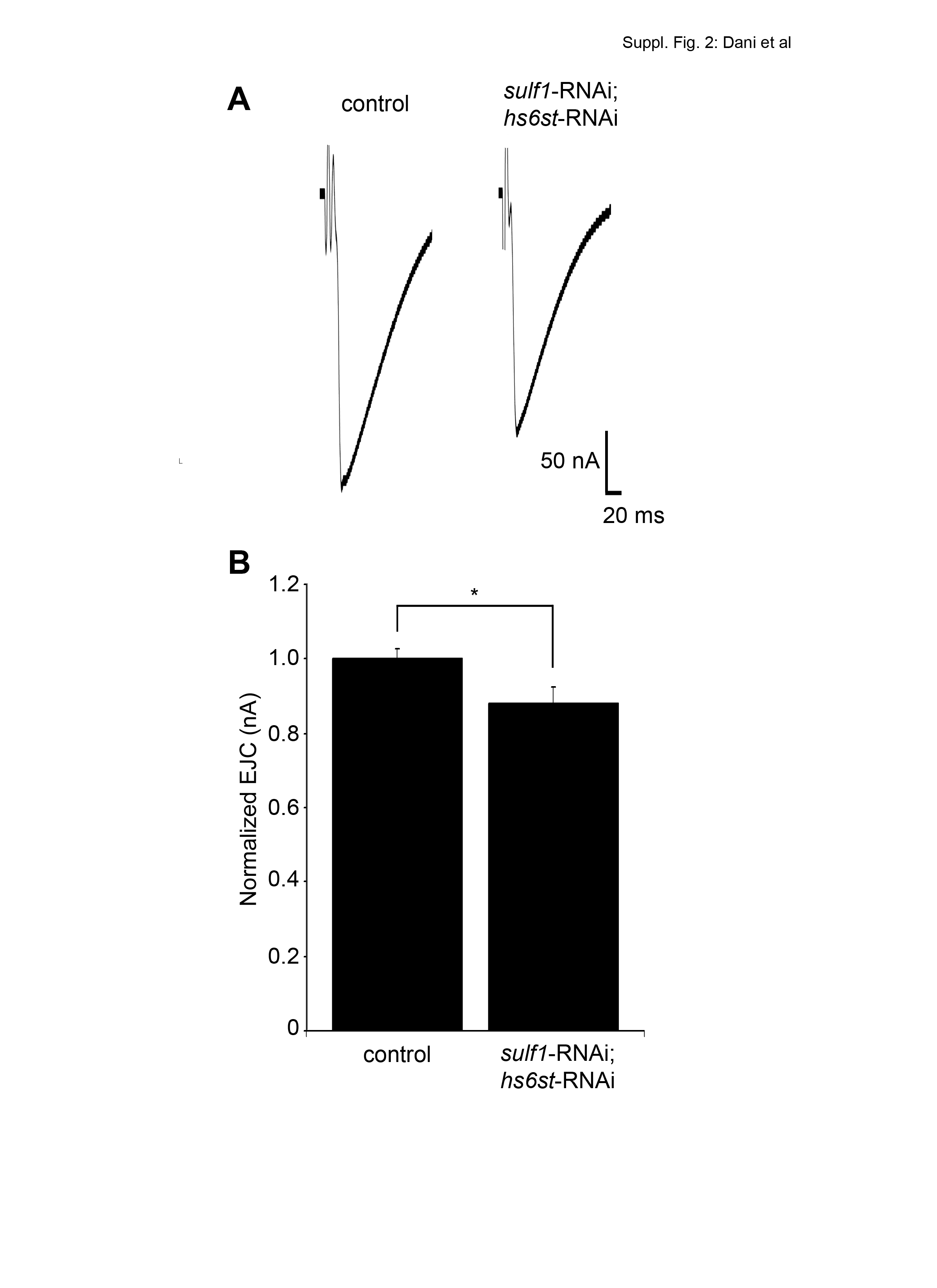

Supplement: Figure S2 — Double knockdown of sulf1 and hs6st measure of EJC amplitude. (A) Representative evoked excitatory junctional current (EJC) traces from control (w1118×UH1-GAL-4) and double knockdown with both sulf1 and hs6st RNAi transgenic lines (UH1-GAL4×UAS-sulf1-RNAi; UAS-hs6st-RNAi). (B) Quantified mean EJC amplitudes (nA) for the two genotypes shown in panel A normalized to control. Sample sizes are ≥12 animals per indicated genotype. Statistically significant differences calculated using student's t-test, * p<0.05, Error bars indicate S.E.M. (TIF) [file pgen.1003031.s002.tif]

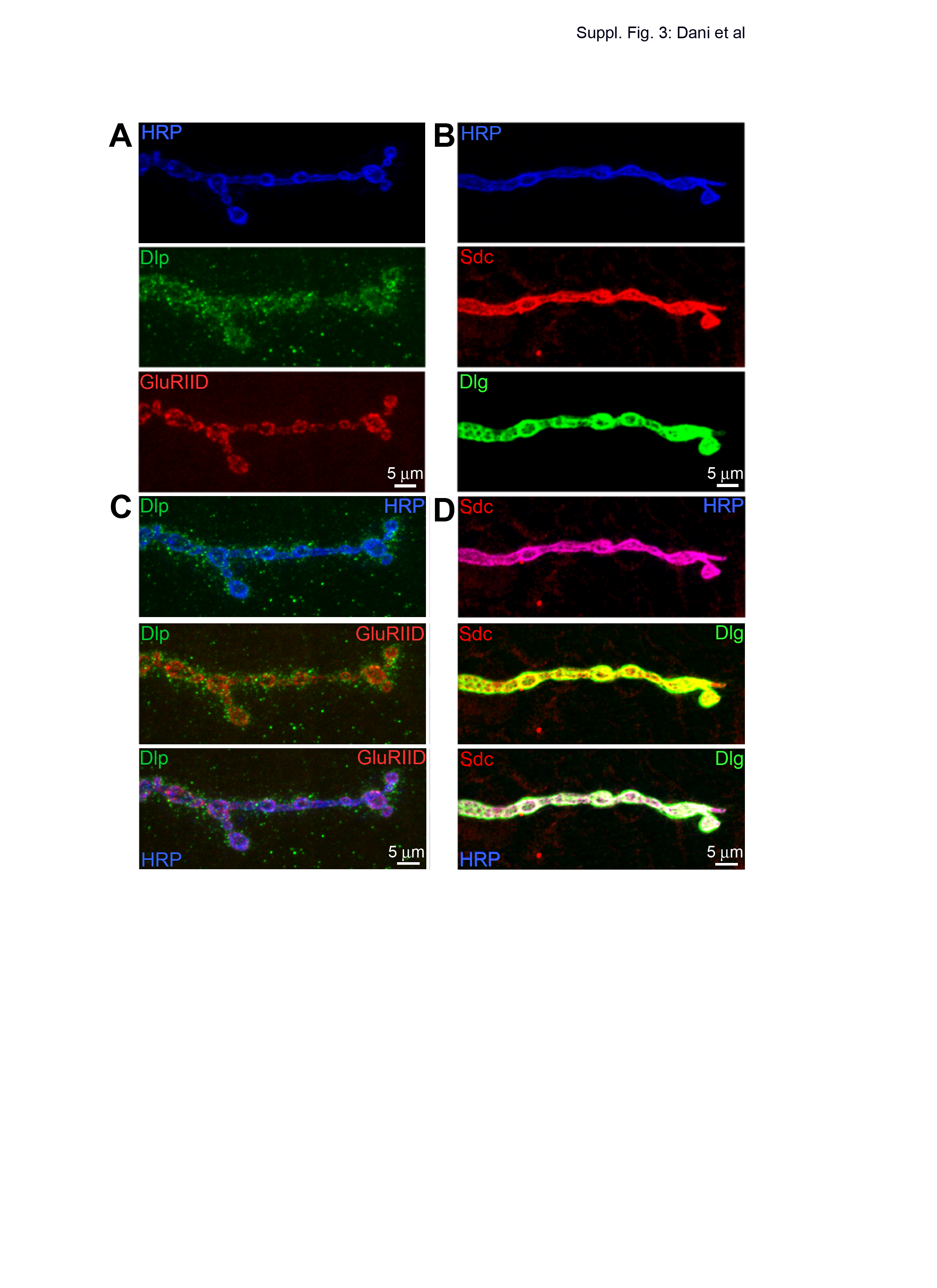

Supplement: Figure S3 — NMJ synaptic localization of Dally-like and Syndecan HSPGs. Representative confocal images showing HSPG synaptic localization at the larval NMJ. (A) Single channel images of presynaptic anti-horseradish peroxidase (anti-HRP, blue), Dally-like Protein (anti-Dlp, green) and postsynaptic glutamate receptor subunit IID (anti-GluRIID, red). (B) Single channel images showing presynaptic anti-horseradish peroxidase (anti-HRP, blue), syndecan (anti-Sdc, red) and postsynaptic Discs Large (anti-DLG, green). (C) Merged image showing Dlp localization with respect to presynaptic HRP, postsynaptic GluRIID and the triple-labeled terminal. (D) Merged image showing Sdc localization with respect to presynaptic HRP, postsynaptic DLG and the triple-labeled terminal. (TIF) [file pgen.1003031.s003.tif]

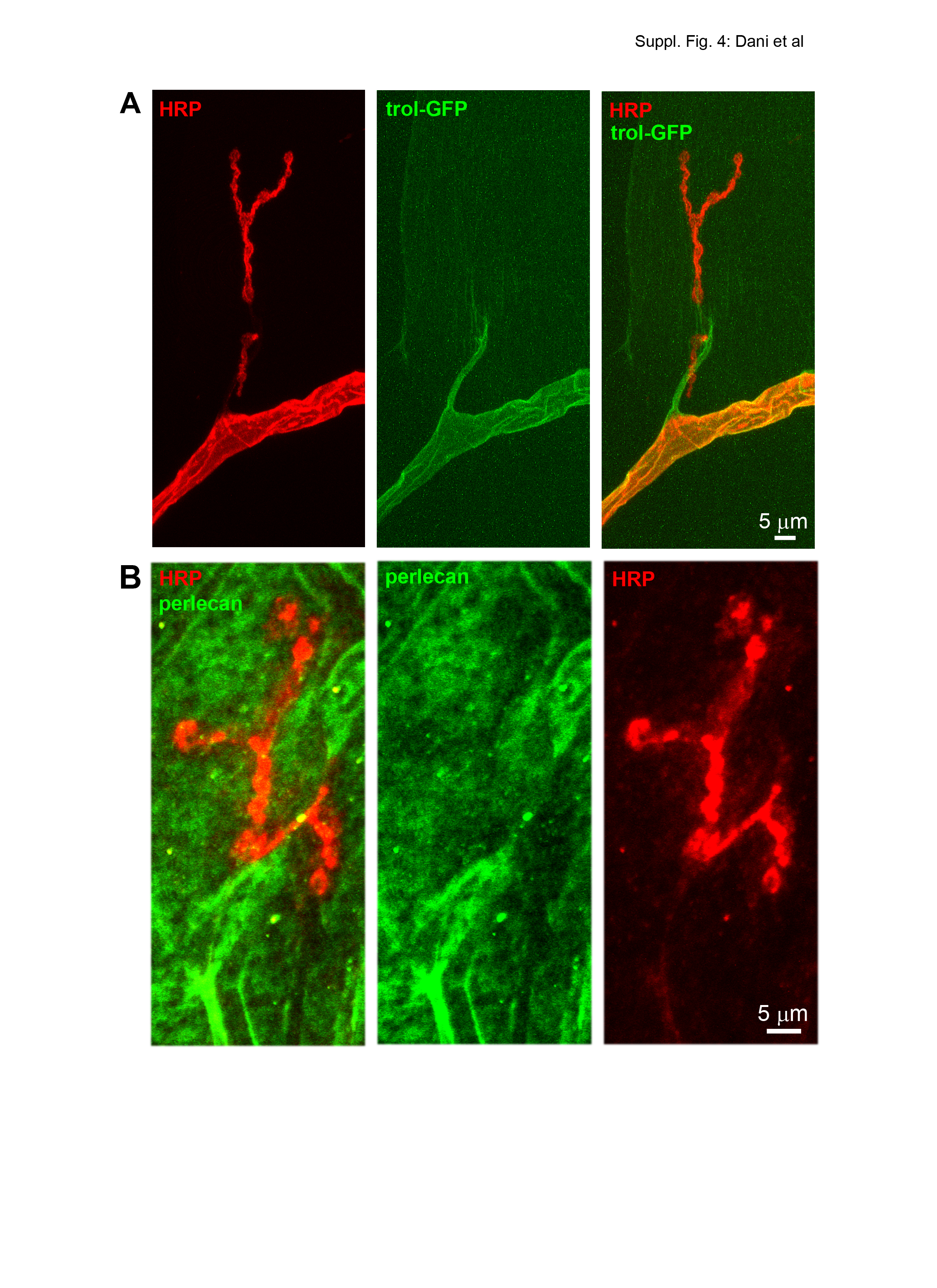

Supplement: Figure S4 — HSPG Perlecan (Trol) is absent from the NMJ synaptic terminal. (A) Representative confocal image showing Perlecan expression at the wandering third instar larval NMJ using the Trol-GFP Flytrap line ZCL1700 from the Flytrap GFP Resource. Single channel and merged images show presynaptic anti-horseradish peroxidase (anti-HRP, red) and Trol-GFP (green). (B) Representative confocal image showing Perlecan (anti-PcanV) antibody staining, shown at a much higher confocal gain than in A to emphasize muscle expression. Perlecan is strongly expressed in the motor nerve, and clearly present on the muscle surface, but is never detectably enriched at the NMJ terminal. In many cases, as in the example shown, Perlecan appears at lower levels in the perisynaptic region surrounding the NMJ than elsewhere on the muscle. (TIF) [file pgen.1003031.s004.tif]

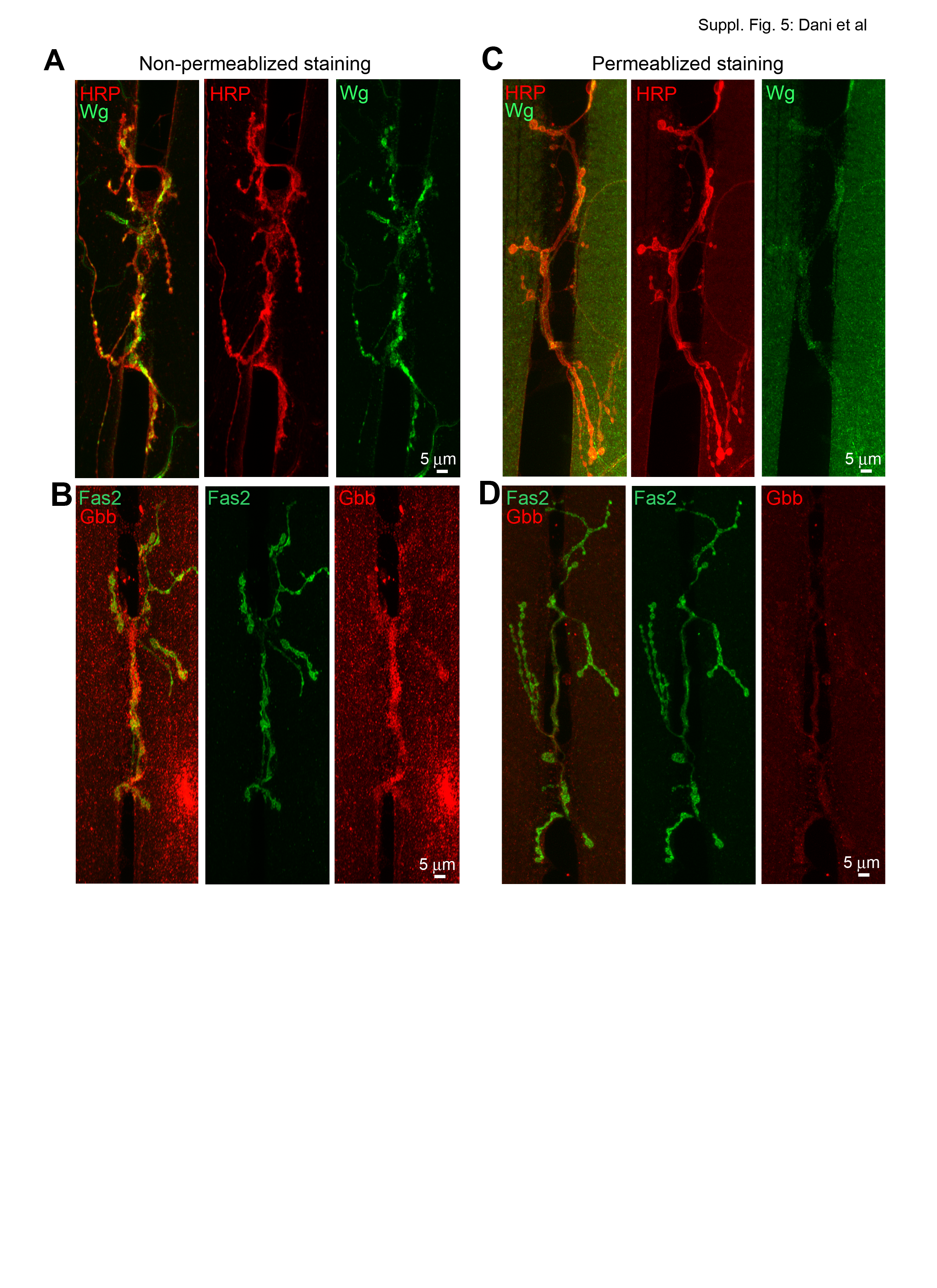

Supplement: Figure S5 — Permeabilized versus non-permeabilized Wg and Gbb labeling. Representative NMJ images of muscle 6/7 in segment A3 from the wandering third instar. Merged and single channel images of (A) anti-horseradish peroxidase (HRP; red) and anti-Wingless (Wg; green), and (B) anti-Fasciclin II (FasII; green) and anti-glass bottom boat (Gbb; red), in non-permeablized labeling conditions in the absence of detergent. Note strong localization of both Wg and Gbb at the NMJ terminal. Merged and single channel images of (C) anti-HRP (red) and anti-Wg (green), and (D) anti-FasII (green) and anti-Gbb (red) in permeablized labeling conditions with 4% paraformaldehyde added to all antibody incubations. Note that most of the synaptic localization of Wg and Gbb is lost. (TIF) [file pgen.1003031.s005.tif]

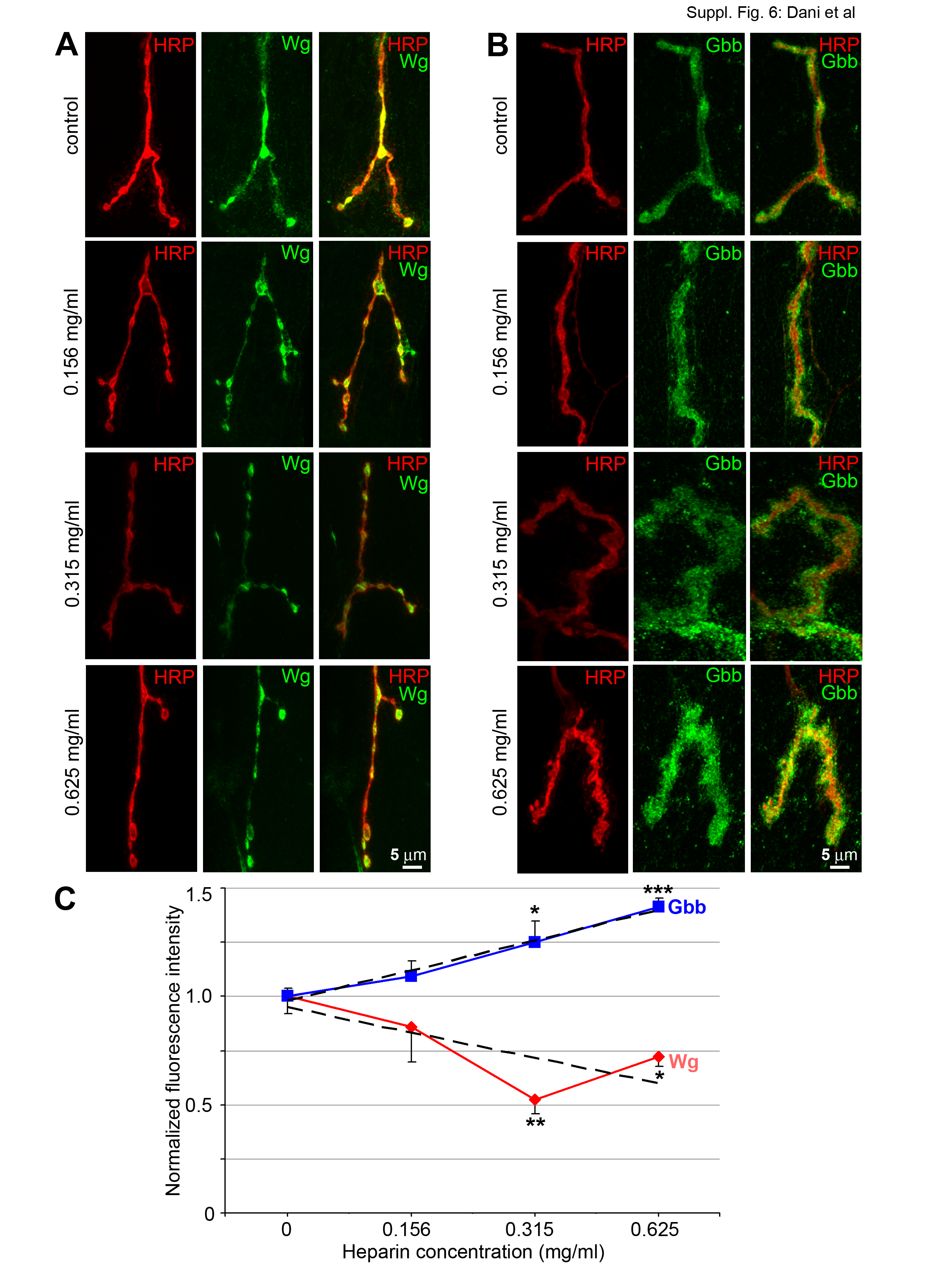

Supplement: Figure S6 — NMJ retention of Wg/Gbb altered by highly-sulfated heparin. Confocal imaging of Wg and Gbb trans-synaptic ligand abundance at the wandering third instar NMJ (muscle 4, segment A3) following acute incubation with highly-sulfated heparin. (A) Single channel and merged images of anti-horseradish peroxidase (HRP; red) and anti-Wingless (Wg; green) following control (no heparin), 0.156 mg/ml, 0.315 mg/ml and 0.625 mg/ml heparin treatments. (B) Single channel and merged images of anti-HRP (red) and anti-glass bottom boat (Gbb; green) following control, 0.156 mg/ml, 0.315 mg/ml and 0.625 mg/ml heparin treatments. (C) Quantification of fluorescence intensity of Wg and Gbb normalized to the internal HRP co-label for the control and indicated heparin concentrations. Individual data points are an average of ≥3 animals. Dotted line shows fitted linear trend lines. Statistically significant differences calculated using student's t-test and indicated as ***p<0.001, ** p<0.01, * p<0.05. Error bars indicate S.E.M. (TIF) [file pgen.1003031.s006.tif]

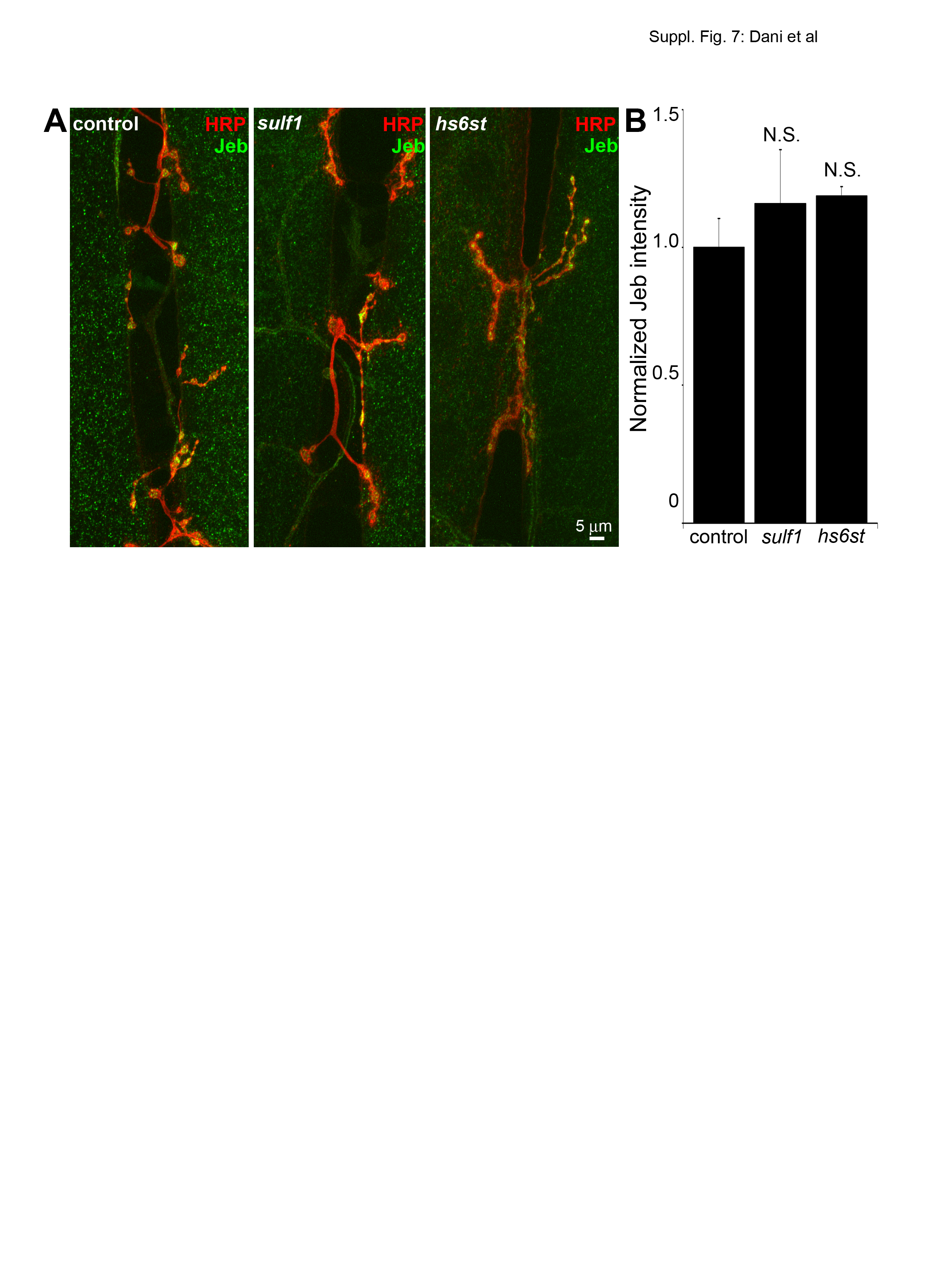

Supplement: Figure S7 — NMJ expression of Jeb ligand unchanged in sulf1/hs6st nulls. (A) Representative NMJ images at the wandering third instar NMJ on muscle 6 in segment A3 from control (w1118), sulf1 and hs6st nulls, labeled with neural marker anti-horseradish peroxidase (HRP; red) and anti-Jelly belly (Jeb; green). Merged images show Jeb tightly localized at synaptic boutons. (B) Quantification of anti-Jeb mean fluorescence intensity levels normalized to HRP co-label and the genetic control. Sample sizes are ≥8 animals per indicated genotypes. Statistically significant differences calculated using student's t-test. N.S. indicates no significant difference. Error bars indicate S.E.M. (TIF) [file pgen.1003031.s007.tif]

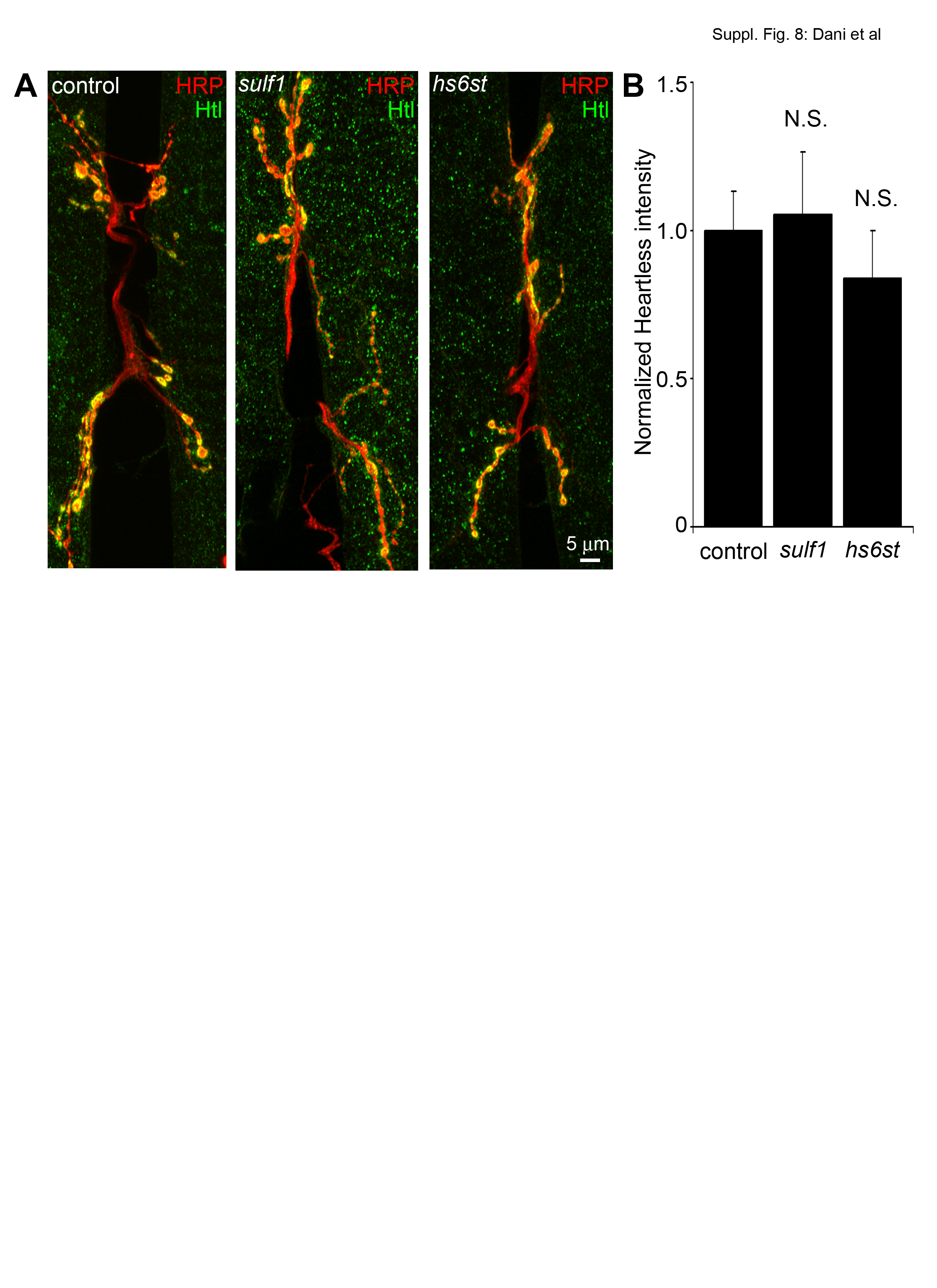

Supplement: Figure S8 — NMJ expression of FGF receptor unchanged in sulf1/hs6st nulls. (A) Representative NMJ images at the wandering third instar NMJ on muscle 6 in segment A3 from control (w1118), sulf1 and hs6st nulls, labeled with neural marker anti-horseradish peroxidase (HRP; red) and anti-Heartless (Htl; green). Merged images show the Htl FGF receptor tightly localized at synaptic boutons. (B) Quantification of Htl mean fluorescence intensity levels normalized to HRP co-label and the genetic control. Sample sizes are ≥7 animals per indicated genotypes. Statistically significant differences calculated using student's t-test. N.S. indicates no significant difference. Error bars indicate S.E.M. (TIF) [file pgen.1003031.s008.tif]

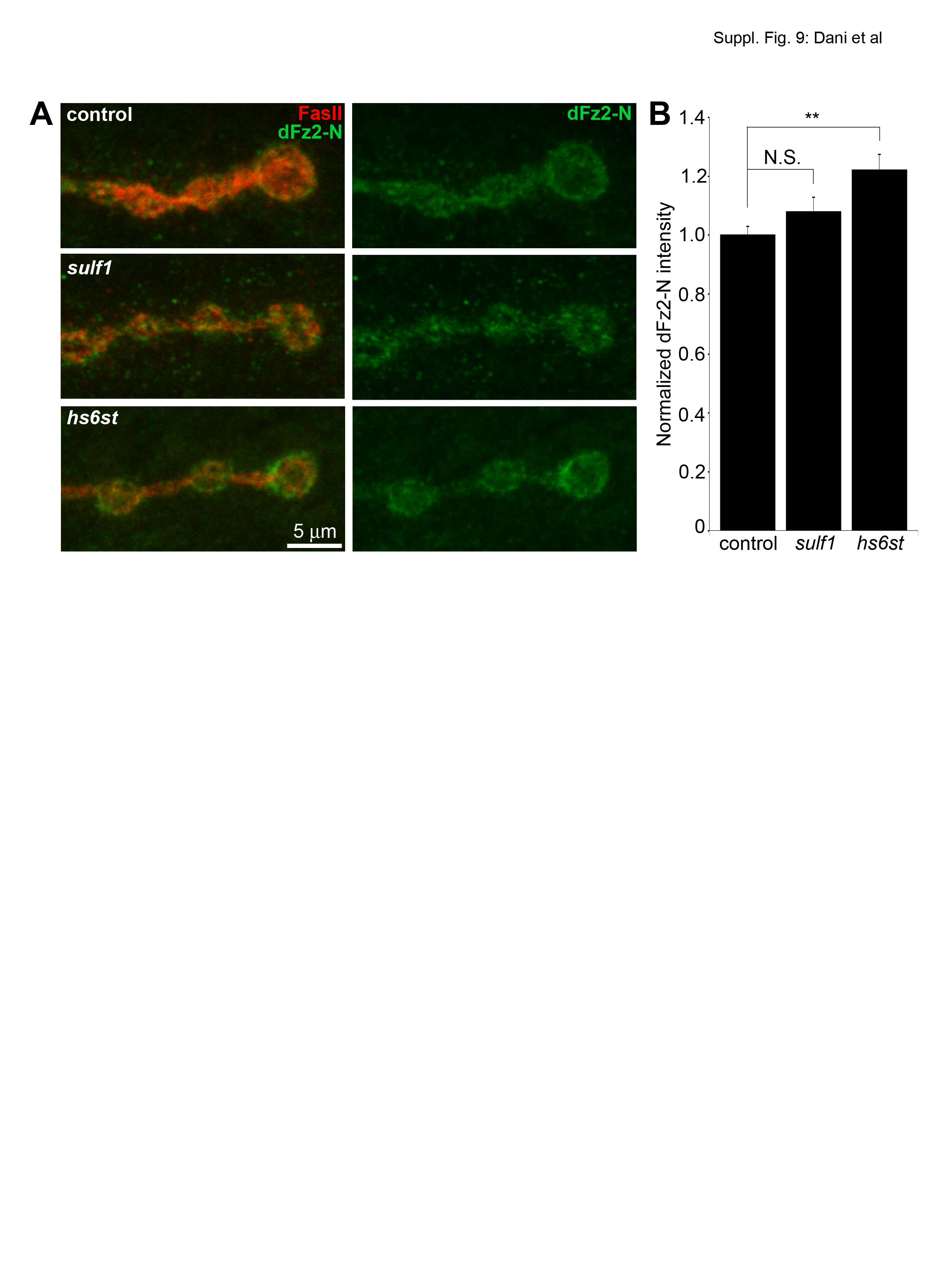

Supplement: Figure S9 — Synaptic Frizzled-2 receptor levels in sulf1 and hs6st nulls. Frizzled-2 receptor N-terminus (dFz2-N) specific antibody shows localized expression surrounding synaptic boutons at the NMJ. (A) Representative wandering third instar NMJ images from muscle 6 in segment A3 for control (w1118), sulf1 and hs6st null mutants, double-labeled with presynaptic neural marker anti-Fasciclin II (FasII, red) and dFz2-N (green). Right: dFz2-N shown alone for clarity. (B) Quantification of dFz2-N mean fluorescence intensity for the indicated genotypes, normalized to the genetic control. Sample sizes are ≥12 animals per genotype. Statistically significant differences calculated using student's t-test, ** p<0.01. Error bars indicate S.E.M. (TIF) [file pgen.1003031.s009.tif]

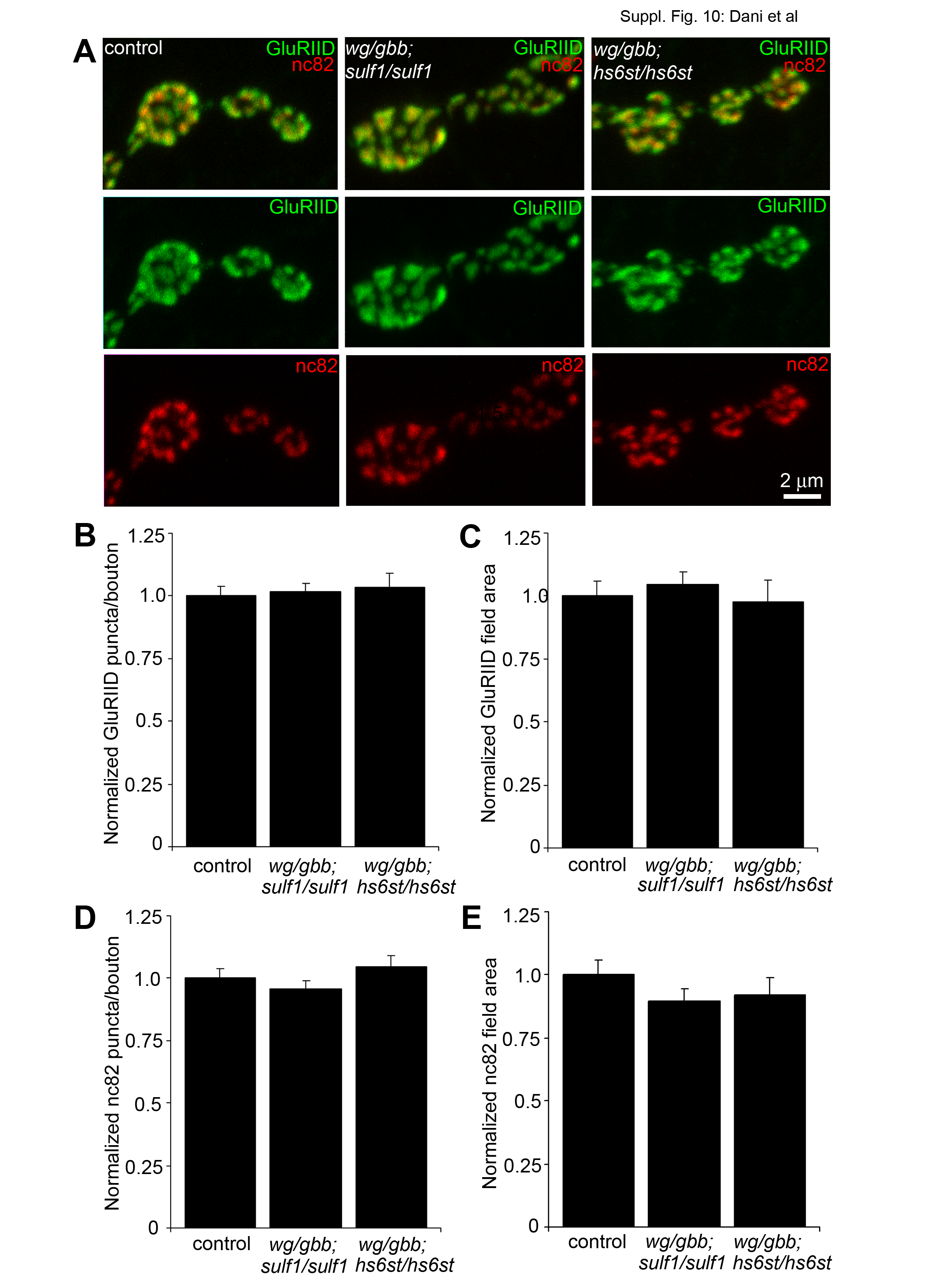

Supplement: Figure S10 — Wg and Gbb signals genetically interact with sulf1 and hs6st nulls. Genetic reduction of Wg and Gbb levels in sulf1 and hs6st homozygous conditions restores molecular synaptic assembly to control levels. (A) Representative NMJ boutons from control (w1118), heterozygous wg/+ and gbb/+ in sulf1 null background (wgI-12/gbb2; sulf1Δ1/sulf1Δ1) and hs6st null background (wgI-12/gbb2; hs6std770/hs6std770) labeled for postsynaptic Bad Reception (Brec) glutamate receptor IID subunit (GluRIID, green) and presynaptic active zone Bruchpilot (anti-nc82, red). Quantification of GluRIID punctae/bouton (B), total GluRIID area (C), Brp punctae/bouton (D) and total Brp area (E), all normalized to the genetic control. All multiply mutant conditions are restored to control levels for all parameters, with no significant differences remaining. (TIF) [file pgen.1003031.s010.tif]

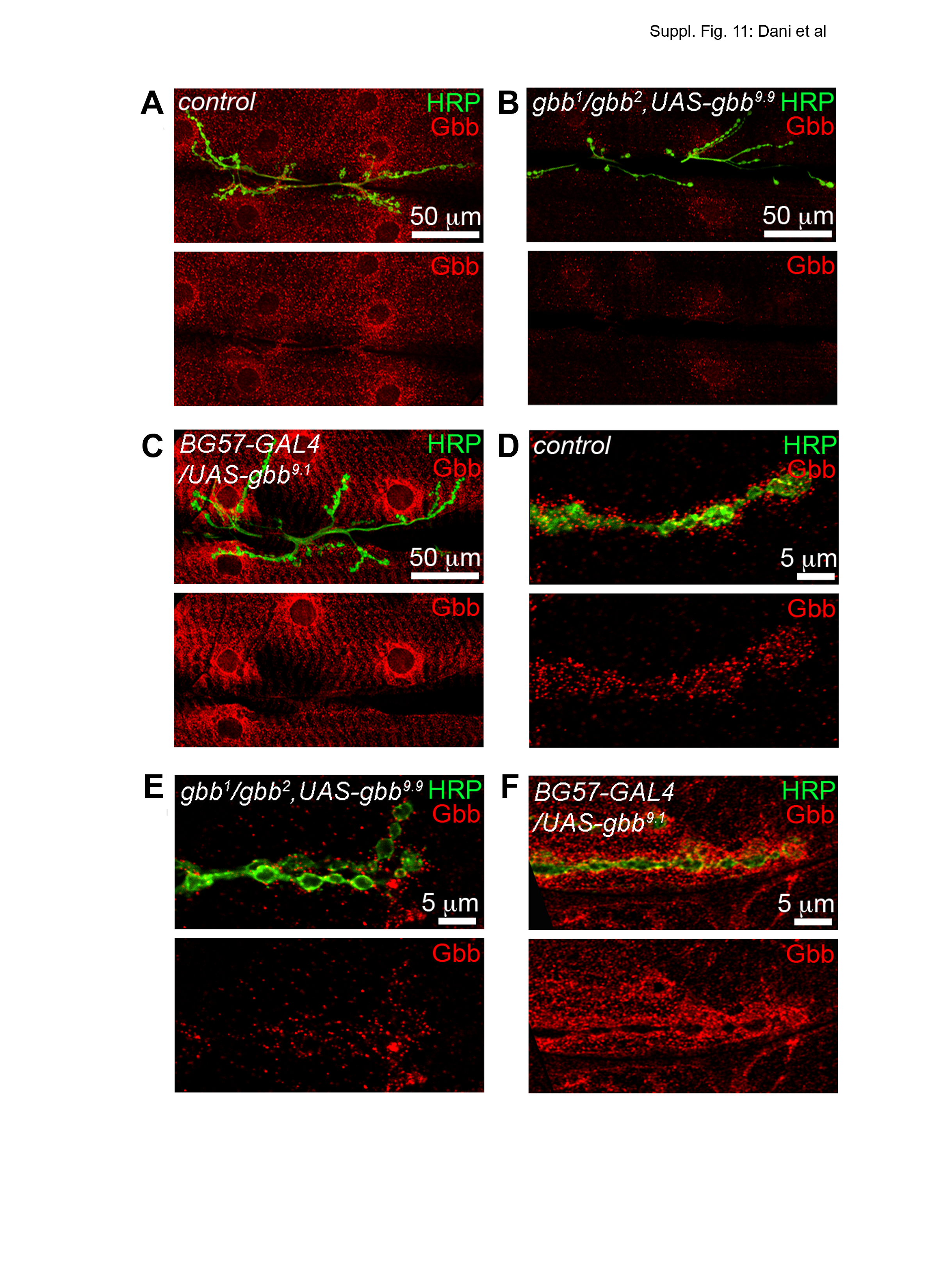

Supplement: Figure S11 — Characterization of anti-Gbb antibody specificity. Representative confocal images of wandering third instar NMJ 6/7 double-labeled with anti-Gbb (red) and anti-HRP (green) under detergent permeabilized (A–C) and non-permeabilized (D–F) conditions. The genotypes analyzed include control (w1118; A,D), gbb1/gbb2,UAS-gbb9.9 (B,E), and BG57-GAL4/UAS-gbb9.1 (C,F). (TIF) [file pgen.1003031.s011.tif]
